# Abnormalities of iron homeostasis and the dopaminergic system in Tourette syndrome revealed by 7T MRI and PET

**DOI:** 10.1093/braincomms/fcaf104

**Published:** 2025-03-10

**Authors:** Dimitrios G Gkotsoulias, Michael Rullmann, Simon Schmitt, Anna Bujanow, Franziska Zientek, Konstantin Messerschmidt, André Pampel, Amira-Philine Büttner, Andreas Schildan, Osama Sabri, Kirsten Müller-Vahl, Henryk Barthel, Harald E Möller

**Affiliations:** Max Planck Institute for Human Cognitive and Brain Sciences, Leipzig 04103, Germany; Department of Nuclear Medicine, Leipzig University Medical Center, Leipzig 04103, Germany; Department of Psychiatry, Social Psychiatry and Psychotherapy, Hannover Medical School, Hannover 30625, Germany; Max Planck Institute for Human Cognitive and Brain Sciences, Leipzig 04103, Germany; Department of Nuclear Medicine, Leipzig University Medical Center, Leipzig 04103, Germany; Department of Nuclear Medicine, Leipzig University Medical Center, Leipzig 04103, Germany; Max Planck Institute for Human Cognitive and Brain Sciences, Leipzig 04103, Germany; Max Planck Institute for Human Cognitive and Brain Sciences, Leipzig 04103, Germany; Department of Nuclear Medicine, Leipzig University Medical Center, Leipzig 04103, Germany; Department of Nuclear Medicine, Leipzig University Medical Center, Leipzig 04103, Germany; Department of Psychiatry, Social Psychiatry and Psychotherapy, Hannover Medical School, Hannover 30625, Germany; Department of Nuclear Medicine, Leipzig University Medical Center, Leipzig 04103, Germany; Max Planck Institute for Human Cognitive and Brain Sciences, Leipzig 04103, Germany; Felix Bloch Institute for Solid State Physics, Leipzig University, Leipzig 04103, Germany

**Keywords:** dopamine D_1_ receptors, Tourette’s syndrome, brain iron, susceptibility MRI, PET

## Abstract

While the implication of a dysfunctional dopaminergic system in Tourette syndrome (TS) is well established, the underlying pathophysiological mechanisms remain unclear. Apart from neurotransmitters, disturbed iron homeostasis and iron regulatory mechanisms are also suspected. Iron is a trace element of fundamental biological importance and is involved in the synthesis and metabolism of dopamine and its receptors and transporters. The goal of the current pre-registered, multi-modal, cross-sectional study was to investigate the relationship between potential iron homeostasis imbalances and dopaminergic system disturbances in patients with TS. Susceptibility-sensitive MRI at 7 Tesla was used to obtain surrogate measures for local brain iron in 25 patients with TS (age 30 ± 9 years, 6 female) and 40 matched control subjects. Additionally, dopamine D_1_ receptor availability was investigated with [^11^C]SCH23390 PET in a subgroup of 20 patients and 20 controls. Significantly reduced sub-cortical magnetic susceptibility, indicating reduced iron levels, was observed in TS patients in the caudate, pallidum, sub-thalamic nucleus, thalamus, red nucleus and substantia nigra. These reductions were accompanied by significant reductions of the [^11^C]SCH23390 binding potential indicating reduced availability of D_1_ receptors in the dorsal striatum. The D_1_ receptor abnormality correlated with tic severity. These results point to alterations of intra-synaptic dopamine release and reduced striatal D_1_ receptor binding, supporting the notion of disruption in multiple functional elements of the dopaminergic system. Such dopaminergic abnormalities appear to be associated with disturbances in iron homeostasis.

## Teaser

Gkotsoulias *et al*. assessed an unexplored facet of Tourette syndrome using 7 Tesla MRI and dopamine D_1_ receptor–specific PET. Reductions in brain iron surrogates and post-synaptic D_1_ receptor availability—also associated with motor tic severity—reveal an inter-play between iron homeostasis and dopaminergic abnormalities, shedding new light on the disorder's complex neurobiological underpinnings.

## Graphical Abstract

**Graphical Abstract:**
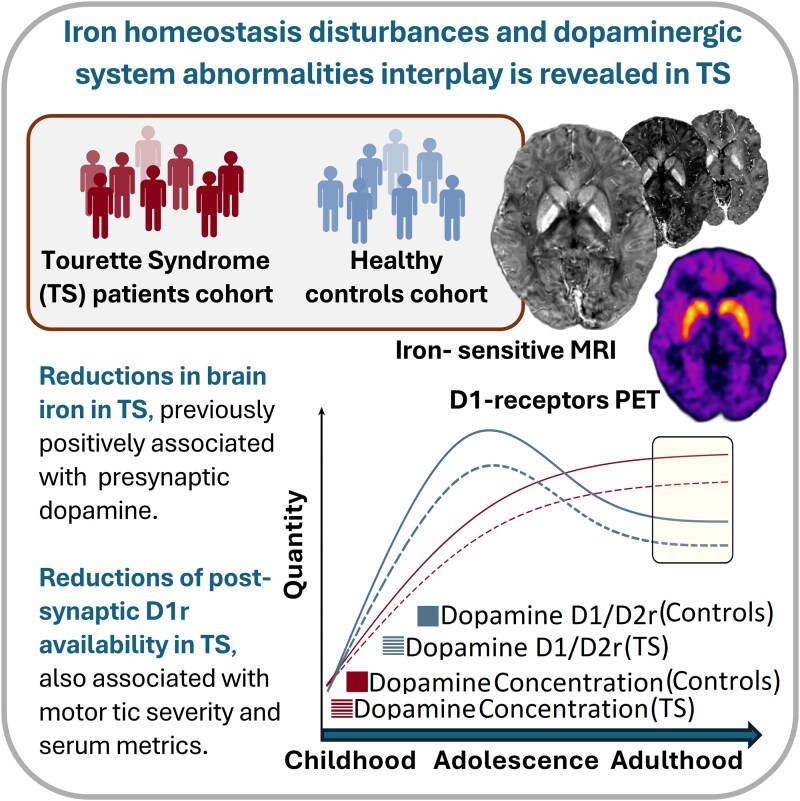

## Introduction

Tourette syndrome (TS) is a neuropsychiatric disorder with a characteristic phenotype, consisting of motor and vocal tics of various severities.^1,2^ Typical are further pre-monitory urges^3^ and the occurrence of comorbid pathologies, such as attention deficit/hyper-activity disorder (ADHD), obsessive compulsive behaviour/obsessive compulsive disorder (OCD), depression and anxiety among others. Alterations in the dopaminergic system involving the cortico-striato-thalamo-cortical and brainstem-striatal circuitries have been identified and linked to phenotypic characteristics, although whether this is the main cause or the outcome of other alterations is still a subject of research.^2,4-6^

PET and MR studies have revealed various differences in D_2_ and D_3_ dopamine receptor availability^7^ as well as alterations in the GABAergic,^8,9^ glutamatergic^5^ and endocannabinoid system.^10,11^ This points to widespread synergistic disturbances in excitatory, inhibitory and modulatory neurotransmitter signaling in the sub-cortex. Most drugs for the treatment of tics are based on dopamine D_2_ receptor antagonists like haloperidol, pimozide, risperidone and aripiprazole.^12^ Recently, the D_1_ receptor antagonist ecopipam also showed significant tic reductions in children and adolescents.^13-15^ While these results point to potential implications of D_1_-type receptors and involvement also of the excitatory pathway in the pathophysiology of TS, potential D_1_ receptor alterations have not yet been assessed by neuroimaging studies.

Apart from neurotransmitter alterations, there is recent evidence of disturbed brain iron homeostasis and iron regulatory mechanisms in TS, based on combined neuroimaging data and normative transcriptomic profiles.^16^ This notion fits with earlier observations indicating reductions of serum ferritin levels in patients with TS.^17-19^ Iron constitutes a trace element of fundamental importance for biological processes, including oxygen transportation, mitochondrial respiration, myelin and neurotransmitter syntheses and more.^20^ Iron homoeostasis is therefore critical for the maintenance of normal brain function, and dysregulation has been consistently associated with various pathologies, including neuropsychiatric disorders.^21-26^

Iron-containing enzymes are involved in the metabolism of dopamine and the production of dopamine receptors and transporters.^20,27-29^ Recent multi-modal imaging studies have linked iron to different aspects of the dopaminergic system (dopamine receptors and transporters) in development and healthy ageing.^30,31^ In these studies, the abundance of iron deposits in the basal ganglia and cortex—quantified by susceptibility-sensitive MRI—was associated with the abundance of pre-synaptic dopamine. Correlations between functional elements of the dopaminergic system and iron storage have been demonstrated in rats, with depleted iron leading to downgraded plateaus of dopamine and dopamine receptor (D_1_, D_2_/D_3_) expressions.^32^

In the current pre-registered, case–control study, PET and MRI were combined to investigate abnormalities of the dopaminergic system and a possible association with brain iron levels in TS. Specifically, we hypothesized that, compared with controls, patients with TS exhibit (i) reduced tonic levels of dopamine and, subsequently, abnormalities in D_1_ receptor availability and (ii) reduced iron stores in sub-cortical structures. To evaluate these hypotheses, we used [^11^C]SCH23390 PET measures of D_1_ receptor availability and iron-sensitive MRI techniques including quantitative susceptibility mapping (QSM) and quantitative imaging of the effective transverse relaxation rate, $R_{2}^{*}=1/T_{2}^{*}$. Serum iron markers, such as ferritin and transferrin levels, were also analysed. Compared with previous research, a particular focus was on D_1_ receptor binding, which to our knowledge has not yet been studied in patients with TS *in vivo* and only sparsely post-mortem.^33,34^ Therefore, this complements previous research, which has largely focused on D_2_-type receptors. The MR aspect was novel in the use of 7T for improved sensitivity and methodologies to separate paramagnetic contribution paramagnetic component of susceptibility (PCS) from diamagnetic contribution diamagnetic component of susceptibility (DCS) to the bulk magnetic susceptibility Δχ. This improved specificity for iron. Assuming that the paramagnetic contributions in brain tissue are primarily associated with the presence of iron, we expected that the PCS would provide more accurate data on iron accumulation than standard QSM.

## Materials and methods

Both aspects of the study were pre-registered (ClinicalTrials.gov IDs NCT05232955 and NCT05233306) and approved by the Ethics Committee at the Medical Faculty of Leipzig University (Approval No. 335/20-ek). Data were acquired from January 2022 to March 2023. All participants were individually informed and signed a written consent form prior to the start of the measurements. They received financial compensation for their participation.

### Population samples for MRI and PET

Twenty-five patients with TS (age: 30.1 ± 8.7 years, 6 females) and 40 age- and sex-matched control subjects (age: 30.6 ± 6.6 years, 8 females; *t* = −0.69, *P* = 0.49) were recruited for MRI acquisitions at 7T at the Max Planck Institute for Human Cognitive and Brain Sciences. In the end, imaging data from 22 patients and 35 controls were included in the final analysis. Three controls were excluded due to study contraindications (alcohol/drug consumption), two controls and two patients due to insufficient image quality (movement artefacts) at visual assessment and one patient due to the inability to undergo the measurement. For PET measurements at 3T, at the Department of Nuclear Medicine, Leipzig University Hospital, 20 patients with TS (age: 28.4 ± 8.9 years, 6 females) and 20 matched controls (age: 28.6 ± 6.2 years, 5 females; *t* = −0.08, *P* = 0.93) were recruited. Of these subjects, 19 patients and 17 controls also participated in the 7T MRI part of the study.

All patients had discontinued any relevant medication that could have an influence on dopaminergic pathways (i.e. antipsychotics, antidepressants) at least 30 days before the measurement. Blood samples were collected from all subjects directly before the imaging experiment. All patients underwent a thorough clinical assessment for TS and comorbidities by trained psychologists of the Department of Psychiatry, Social Psychiatry and Psychotherapy, Hannover Medical School. The assessments included Yale Global Tic Severity Scale (YGTSS),^35^ the most important and widely accepted tic severity metric; Adult Tic Questionnaire (ATQ),^36^ a tic self-rating scale; Pre-monitory Urge for Tics Scale (PUTS),^37^ a rating of the pre-monitory urges for tics; Clinical Global Impressions–Severity (CGI–S),^38^ a widely used assessment tool to evaluate the overall severity of illness in patients with various psychiatric disorders; Gilles de la Tourette Syndrome–Quality Of Life (GTS–QOL),^39^ a correlation of the quality of life with TS symptomatology; and Pittsburgh Sleep Quality Index (PSQI),^40^ constituting a part of common clinical self-assessments. Comorbidities of TS were evaluated using the Diagnostic and Statistical Manual of Mental Disorders; 4th edition (DSM-IV) symptom list and Conners’ Adult ADHD Rating Scale (CAARS)^41^ for ADHD, Yale-Brown Obsessive Compulsive Scale (Y-BOCS)^42,43^ for OCD, Rage Attack Questionnaire (RAQ)^44,45^ for rage attacks, Beck Anxiety Inventory (BAI)^46,47^ for assessment of anxiety, Beck Depression Inventory, 2nd revision (BDI-II)^48,49^ for depression and Autism-spectrum Quotient questionnaire, short version (AQ-k)^50^ for autistic symptoms. Patients with severe head tics were excluded to ensure suitable conditions for MRI and PET. Results from the clinical assessments are summarized in Table 1.

**Table 1:** **Results of the clinical assessments of the patients** (***n* = 24 for clinical tests and *n* = 22 for self-assessment tests)**

Test	Mean ± SD
**Tics**	
YGTSS	
Motor tics	14.6 ± 3.8
Vocal tics	10.1 ± 5.8
Total	24.3 ± 7.8
ATQ: motor tics	
Frequency	1.23 ± 0.70
Intensity	0.87 ± 0.41
ATQ: vocal tics	
Frequency	0.67 ± 0.62
Intensity	0.50 ± 0.39
PUTS	22.1 ± 6.1
Quality of life	
GTS–QOL	17.6 ± 12.1
Comorbidities	
DSM-IV	5.0 ± 4.6
CGI–S	4.08 ± 0.93
CAARS	50.2 ± 20.9
PSQI	13.0 ± 7.1
Y-BOCS	8.7 ± 10.8
RAQ	11.2 ± 10.6
BAI	6.7 ± 6.9
BDI-II	9.8 ± 6.4
AQ-k	55.3 ± 7.5

SD, standard deviation.

### 7T MRI measurements

Experiments at 7T were performed on a MAGNETOM Terra (Siemens Healthineers, Erlangen, Germany) using a circularly polarized transmit, 32-channel receive array coil (Nova Medical, Wilmington, MA, USA). Structural Magnetization Prepared 2 Rapid Acquisition Gradient Echoes (MP2RAGE)^51^ scans were acquired with 1 mm isotropic nominal resolution; a field of view (FOV) of 228×228×208 mm^3^ (sagittal slice orientation, anterior-to-posterior phase encoding); flip angles, α_1_ = 5°, α_2_= 6°; repetition time (TR) = 4300 ms; echo time (TE) = 2.9 ms; inversion times (TI) = 840 and 2370 ms; bandwidth (BW) = 130 Hz/pixel and GeneRalized Autocalibrating Partially Parallel Acquisitions (GRAPPA)^52^ acceleration factor 3 and a partial-Fourier^53^ factor *f_p_* = 6/8 in phase-and slice-encoding directions. The acquisition time (TA) was ≈6 min. Susceptibility-sensitive Multi-Echo Gradient-Recalled Echo (ME-GRE)^54^ acquisitions (TA≈14:30 min) were performed after individual manual shimming with a nominal isotropic resolution of 0.8 mm (transverse orientation, left-to-right phase encoding); FOV = 266 × 266 × 214 mm^3^; *α* = 12°; TR = 47 ms; nine echoes with first TE = 5.03 ms and echo spacing of 4.1 ms; BW = 590 Hz/pixel; GRAPPA factor 3; and *f_p_* = 7/8 in phase-encoding direction.

### PET measurements

PET acquisitions using the D_1_-specific radiotracer [^11^C](*R*)-2,3,4,5-tetrahydro-8-chloro-3-methyl-5-phenyl-1H-3-benzazepine-7-ol ([^11^C]SCH23390)^55,56^ were obtained on a Biograph mMR hybrid PET/3T MRI scanner (Siemens Healthineers, Erlangen, Germany) with a head matrix coil specialized for low attenuation profiles. After acquisition of a localizer, the radiotracer was intravenously administered as a bolus over 90 s (mean radioactivity 483 ± 30 MBq), and a 90-min dynamic PET scan (23 frames: 4 × 15, 4×60, 5×120, 5 × 300, 5 × 600 s; reconstructed spatial resolution 1 × 1 × 2 mm^3^) was performed simultaneously with MRI. Structural MP2RAGE data were recorded with 1 mm isotropic nominal resolution; FOV 256 × 256 × 176 mm^3^ (sagittal orientation, anterior-to-posterior phase encoding); α_1_ = 5°, α_2_ = 6°; TR = 5000 ms; TE = 2.98 ms; TI = 720 and 2500 ms; BW = 240 Hz/pixel; GRAPPA acceleration factor *R* = 2 and TA≈12 min. Also included were a 10-min resting-state functional MRI (fMRI) scan to assess head movements and MR-based attenuation correction.^57^

### Processing of susceptibility-sensitive MRI

The complex-valued GRE data from each coil channel (*n* = 32) were saved individually and adaptively combined offline (with preservation of the phase information), along with Laplacian phase unwrapping.^58,59^ FSL^60^ was used for background masking, variable-kernel sophisticated harmonic artefact reduction for phase data for background-field removal^58,61,62^ and an iterative least-squares solver in Matlab for field-to-source inversion.^58,63^ The phase data from the first five echoes were used for QSM reconstruction. The last four echoes indicated a reduced signal-to-noise ratio and phase unwrapping-related artefacts, which are often observed at long TE.^64^ For quantification of Δχ, the maps were referenced to the average value in the CSF, measured within a subject-specific manually segmented region of interest (ROI) in the ventricles.^65^ The Auto-Regression on Linear Operations algorithm^66^ was used with the first six echoes of the ME-GRE magnitude volumes for voxel-wise fitting of $R_{2}^{*}$. Visual inspection showed no evident advantage (e.g. improved indication of the noise floor) of using further echoes in the fit. The estimations of Δχ and $R_{2}^{*}$ were finally fed into the DiamagnEtic COMponent and Paramagnetic cOmponent Separation (DECOMPOSE) model for separation of PCS from DCS.^67^ All calculated maps were individually assessed for quality and potential artefacts. A schematic illustration of the entire processing pipeline is shown in Supplementary Fig. 1.

### Processing of D_1_ receptor–sensitive PET

The PET volumes were normalized based on the attenuation correction and the exact radioactivity at the onset of the measurement. Dynamic PET data were registered to the 3T MP2RAGE data and motion corrected in PMOD (version 3.5, PMOD Technologies LLC, Fällanden, Switzerland). No volumes were identified as outliers. Estimations of frame-wise displacements from the resting-state fMRI acquisition indicated one subject with displacements above a commonly used threshold of 0.25 mm,^68^ which was excluded. Another patient had to be excluded due to a failure of the PET reconstruction. Parametric images of the [^11^C]SCH23390 non-displaceable binding potential ($BP_{\mathrm{ND}}$; a combined measure of the density of available receptors and the affinity of the radioligand) were generated in PMOD from the PET data by the Multilinear Reference Tissue Model 2 with two parameters and the cerebellar cortex as the reference tissue.^69^ The resulting parametric 3D maps were then registered with the 3T MP2RAGE data, resulting in fused PET and MP2RAGE volumes of 1 mm isotropic nominal resolution, which were eventually used in all further analyses.

### Segmentation of deep grey matter nuclei

Segmentations were performed in the 7T ME-GRE native space to avoid loss of accuracy of the iron surrogate metrics due to registration processes.^16^ After skull-stripping, the T_1_-weighted ‘uniform’ MP2RAGE volume^51^ was registered to a selected bias field-corrected GRE magnitude image using Advanced Normalization Tools (ANTs) in Python (https://github.com/ANTsX/ANTs). As the volumes were acquired at different spatial resolutions, the registration process started with an initial affine transformation, followed by a non-linear step using mutual information for optimization (ANTs symmetric normalization protocol).^70^ The quality of registration was visually assessed for each participant. Based on the registered MP2RAGE volume, FSL FIRST^71^ was used for automated segmentations of the caudate, putamen, pallidum and thalamus. All masks underwent erosion by two voxel layers to avoid inclusion of neighbouring regions, which—especially around the basal ganglia—considerably differ in their susceptibility properties. For example, confounding diamagnetic contributions of strongly myelinated white-matter (WM) fibres of the internal capsule to the paramagnetic, iron-rich pallidum or partial voluming at interfaces between ventricular CSF and surrounding tissue must be minimized.

While major sub-cortical nuclei can be automatically delineated based on the MP2RAGE ‘uniform’ contrast, the brainstem nuclei are notoriously difficult to localize on such images, and T_2_*-weighted or QSM data have been used for that purpose. Multiple studies have further proposed automated methods based on a combination of contrasts, machine learning, Bayesian approaches and atlases.^16,72-74^ In this work, QSM maps were used for manual delineation of the substantia nigra (SN), red nucleus (RN) and sub-thalamic nucleus (StN) on the 7T images, using MRIcron (https://www.nitrc.org/projects/mricron) and previous practices.^75^ The approach is illustrated in Supplementary Fig. 2. Note that the brainstem nuclei tend to have spatial contrast differences within the structure, which may lead to segmentation errors. For example, the QSM contrast in the SN typically indicated a hypo-intense region (Supplementary Fig. 2A) corresponding to Nigrosome 1.^76,77^ It needs to be included in the delineation and can be regarded as the end of the structure. Iron accumulation in brainstem nuclei can further increase the contrast with neighbouring regions, inducing a tendency to over-estimate the regions. Therefore, erosion was always applied to the manually extracted segmentation masks.

### Statistical analysis

To account for inhomogeneous variance, group differences for each sub-cortex ROI were assessed using Welch's *t*-test (single-sided for Δχ, PCS and $R_{2}^{*}$ and two-sided for DCS and PET measures), with significance threshold *P*_FDR_ < 0.05 after false discovery rate (FDR) correction. Cohen‘s *d* and confidence intervals were calculated for a complete overview of the group differences. For the ROIs in the caudate, putamen, pallidum and thalamus, the same analysis was performed for the PET images (registered to ME-GRE native space). Group differences in the serum metrics total iron, ferritin (Ft), transferrin (Tf), transferrin saturation and soluble transferrin receptors were assessed in the same way, after removal of outliers, which were defined as values 1.5 times the inter-quartile range above the third or below the first quartile of the distribution.^78^ The strengths of correlations were assessed by Pearson's correlation coefficients *R*. For investigating correlations between the different iron surrogate metrics (Δχ, PCS and $R_{2}^{*}$), the corresponding values for controls and patients were assessed together. To analyse relations between MRI and PET results, the metrics were split into those from control subjects and patients.

## Results

**Figure 1:**
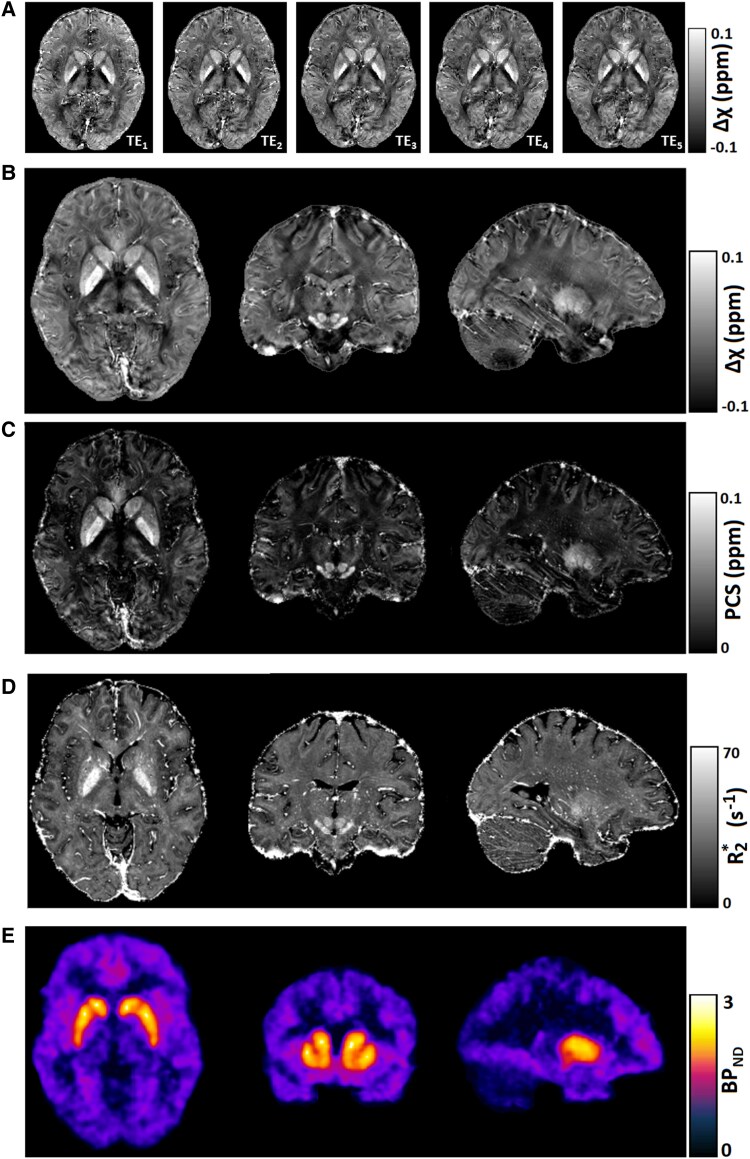
**Examples of susceptibility-sensitive MRI and D_1_ receptor availability PET maps obtained in a randomly selected subject.** (**A**) QSM estimates of the first five echoes acquired at echo times $TE_{1}$, …, $TE_{5}$, which were subsequently combined for the final QSM reconstruction of Δχ. (**B**) Axial, coronal and sagittal planes of the final QSM result. (**C**) Corresponding PCS results obtained with DECOMPOSE. (**D**) Corresponding $R_{2}^{*}$ maps. (**E**) Example of a [^11^C]SCH23390 $BP_{\mathrm{ND}}$ map.

Examples of susceptibility-sensitive images including quantitative maps of Δχ, $R_{2}^{*}$ and PCS from a random participant in our study are presented in Fig. 1. All parameters show elevated intensities in the basal ganglia, with clear differentiation from surrounding WM structures. Due to a dominating DCS, WM appears with low intensity on the PCS map. An [^11^C]SCH23390 $BP_{\mathrm{ND}}$ map obtained with PET is also included as Fig. 1E. Increased $BP_{\mathrm{ND}}$ in the basal ganglia, especially in the putamen and pallidum, is clearly evident, in line with areas of known high D_1_ receptor density.^56,79-82^

### Blood iron measures and MRI-derived brain iron surrogates

Blood transferrin levels were significantly reduced in patients with TS compared with controls (2.34 ± 0.18 versus 2.56 ± 0.29 g/l, *t* = −3.30, *P* = 0.001). The other parameters did not show significant differences (serum iron: 17.0 ± 3.2 versus 17.7 ± 5.0 µmol/l, *t* = −0.16, *P* = 0.86; serum ferritin: 94 ± 53 versus 95 ± 67 ng/ml, *t* = −0.25, *P* = 0.79; transferrin saturation: 28.6 ± 6.5% versus 27.2 ± 7.8%, *t* = 0.75, *P* = 0.45 and soluble transferrin receptors: 2.68 ± 0.35 versus 2.69 ± 0.41 mg/l, *t* = 0.47, *P* = 0.23). Serum ferritin is known to be affected by dietary habits, which may have biased the result.^83^ Retrospectively collected information indicated that a number of participants with ferritin levels towards the lower end of the normal range had recently adjusted their eating behaviour towards a low-meat, vegetarian or vegan diet. After excluding these subjects in an explorative analysis, a significantly lower serum ferritin level was obtained in patients compared with controls (95 ± 53 versus 126 ± 88 ng/ml, *t* = −1.58, *P* = 0.05; Supplementary Fig. 3). However, this explorative analysis remains incomplete, as information about dietary habits of the full cohorts was not obtained.

**Figure 2:**
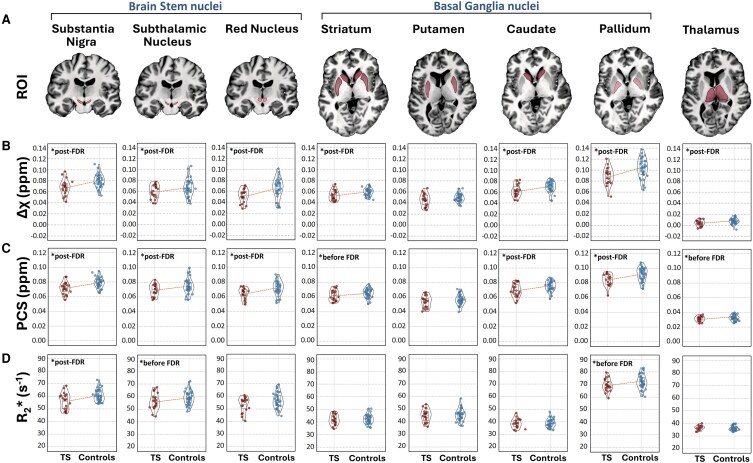
**Sub-cortical MRI-derived brain iron surrogates.** Shown are corresponding ROIs indicated on an axial slice of the fourth echo of the ME-GRE volumes (**A**) as well as violin plots of regional Δχ (referenced to CSF) (**B**), PCS (**C**) and $R_{2}^{*}$ (**D**) differences between patients with TS and control subjects. Graphs marked with an asterisk illustrate significant reductions in the patient group (*P* < 0.05) after single-sided Welch's *t*-test, as well as those that survived FDR correction (*P*_FDR_ < 0.05). All statistical results correspond to sample sizes of *n* = 21 patients with TS (red symbols) and *n* = 35 control subjects (blue symbols).

All MRI-derived brain iron surrogates indicated common significant reductions in most sub-cortex ROIs in patients with TS compared with controls. Supplementary Table 1 summarizes these results. Δχ was significantly reduced in patients compared with controls with medium or large effect sizes (Cohen's *d* > 0.5 or >0.8, respectively) in the SN, StN, RN, striatum, pallidum and thalamus (Fig. 2A and B). Within the striatum, Δχ was significantly reduced in the caudate, whereas the reduction in putamen did not reach significance. It is expected that the DECOMPOSE-QSM methodology depicts the brain iron distribution clearer than standard QSM due to a separation of diamagnetic from paramagnetic contributions. Consistently, the PCS maps showed reductions in patients compared with controls as found with Δχ, with reduced error probabilities (but roughly unchanged effect sizes) in the SN, StN, RN and pallidum (Fig. 2C). These nuclei are known to be particularly rich in iron.^84-86^ Reductions in striatum and thalamus were insignificant after FDR correction. Finally, the general observations were also reproduced on $R_{2}^{*}$ maps (Fig. 2D), albeit with lower statistical power (i.e. reductions in StN, RN and pallidum did not reach significance after FDR correction, and the effect sizes were small or very small). Note that statistically superior results from QSM compared with $R_{2}^{*}$ in neurodegenerative diseases were also reported previously.^87^

**Figure 3:**
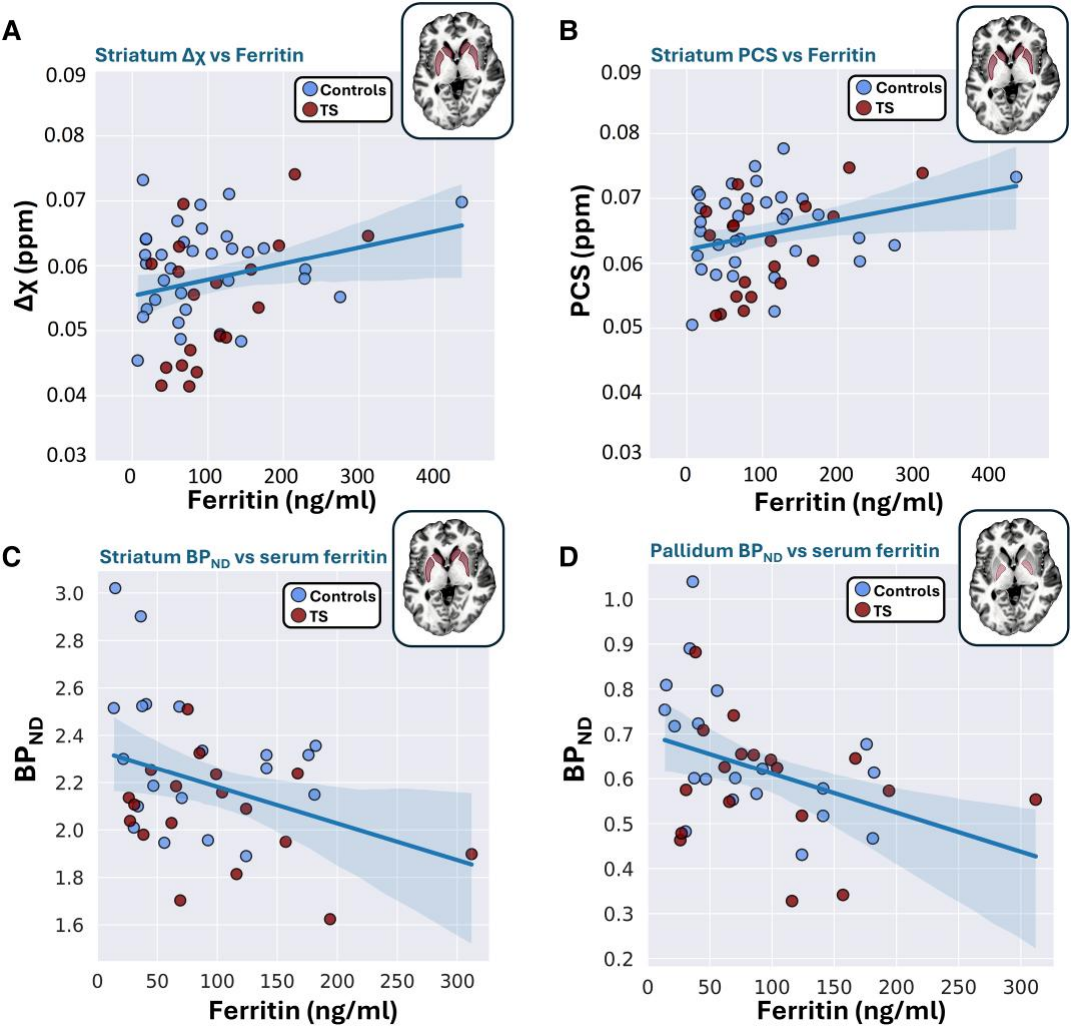
**Linear relations y=ax+b of MRI-derived brain iron surrogates or PET-based D_1_ receptor availability and serum ferritin levels.** Variation of (**A**) Δχ [*a* = 2.5 × 10^–5^ ppm/(ng/ml), *b* = 0.055 ppm; *R* = 0.41, *P* = 0.0024] and (**B**) PCS [*a* = 2.2 × 10^–5^ ppm/(ng/ml), *b* = 0.062 ppm; *R* = 0.35, *P* = 0.0088] in the striatum (putamen and caudate) as well as [^11^C]SCH23390 $BP_{\mathrm{ND}}$ in (**C**) the striatum (*a*= −0.0015, *b*=2.34, *R* = −0.35, *P* = 0.037) and (**D**) the pallidum (*a* = −0.0009, *b* = 0.69, *R* = −0.39, *P* = 0.015) with serum ferritin level in the combined cohort of patients with TS (red symbols; *n* = 21 and 18 for the MRI and PET data, respectively) and control subjects (blue symbols; *n* = 35 and 20, respectively). All correlations were assessed by calculating Pearson's coefficient *R*. Data from one patient was excluded due to a presumably corrupted ferritin measurement. Note the lower scattering of the PCS data compared with Δχ.

Associations between local brain iron surrogates Δχ and PCS in the striatum and the serum ferritin levels are shown in Fig. 3 for the combined cohort of patients and controls. Similar correlations with serum ferritin levels were also observed for Δχ in the StN (Supplementary Fig. 4A; *R* = 0.29, *P* = 0.034) and for PCS in putamen (Supplementary Fig. 4B; *R* = 0.31, *P* = 0.023) as well as for serum transferrin levels and PCS in the pallidum (Supplementary Fig. 4C; *R* = 0.29, *P* = 0.037). Generally, iron surrogate measures in further sub-cortical regions showed similar weak correlations with serum iron metrics, however without reaching significance (Supplementary Table 2).

Brain iron surrogates showed no significant correlations with tic assessment scores (Supplementary Tables 3 and 4).

### PET-derived D_1_ receptor availability

**Figure 4:**
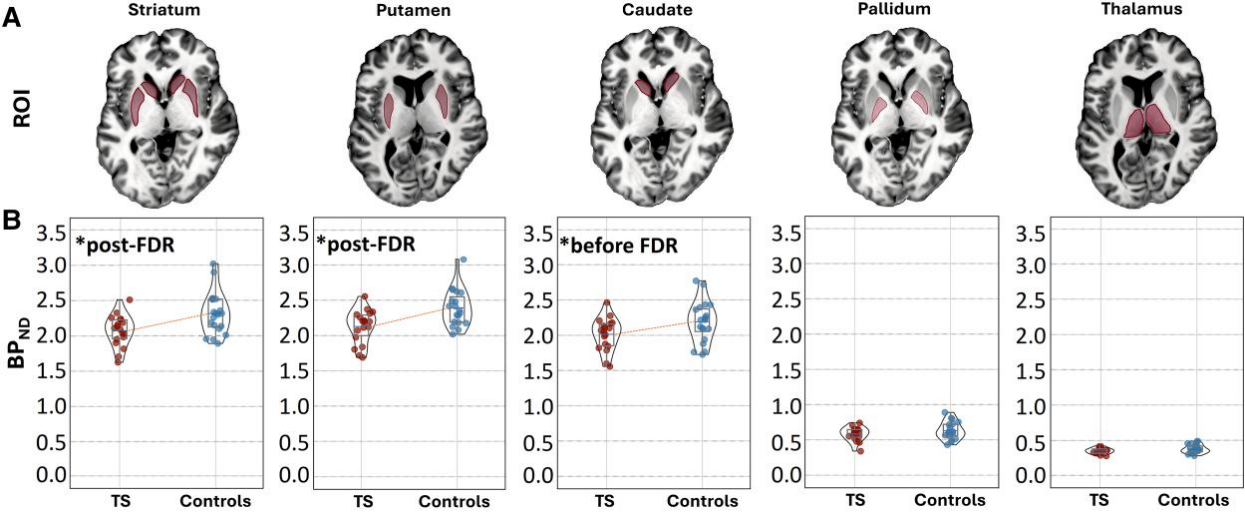
**Sub-cortical D_1_ receptor availability.** Shown are corresponding ROIs indicated on an axial slice of the fourth echo of the ME-GRE volumes (**A**) and violin plots of regional [^11^C]SCH23390 $BP_{\mathrm{ND}}$ in *n* = 18 patients (red symbols) and *n* = 19 control subjects (blue symbols) (**B**). Graphs marked with an asterisk illustrate significant reductions (two-sided Welch's *t*-tests) in the patient group (*P* < 0.05) and those that survived FDR correction (*P*_FDR_ < 0.05).

The analysis of the PET data revealed significant reductions of [^11^C]SCH23390 $BP_{\mathrm{ND}}$ in patients compared with controls in the putamen and trends towards reductions in the caudate and thalamus (Fig. 4; Supplementary Table 1). The BP_ND_ reduction in the pallidum did not reach significance after FDR correction. Other nuclei did not show significant group differences.

**Figure 5:**
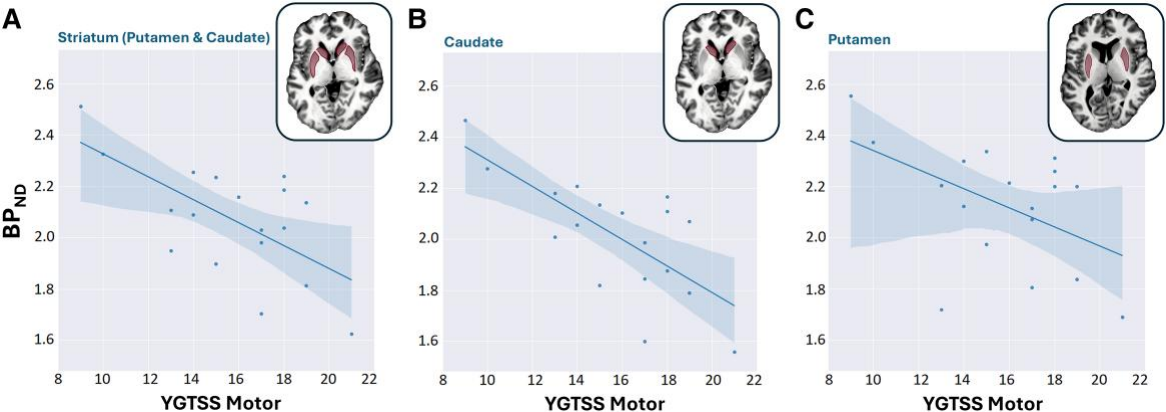
**Association between PET-derived D_1_ receptor availability and tic severity in patients with TS (*n* = 18).** Approximate linear relations y=ax+b of [^11^C]SCH23390 $BP_{\mathrm{ND}}$ and YGTSS motor sub-scores were obtained in (**A**) the striatum (*a* = −0.045, *b* = 2.78; *R* = −0.55, *P* = 0.02), (**B**) caudate (*a* = −0.05, *b* = 2.79; *R* = −0.71, *P* = 0.0015) and (**C**) putamen (*a* = −0.0325, *b* = 2.64; *R* = −0.49, *P* = 0.05). All correlations were assessed by calculating Pearson's coefficient *R*.

The PET-derived [^11^C]SCH23390 $BP_{\mathrm{ND}}$ correlated negatively with the YGTSS motor tic sub-score (i.e. high [^11^C]SCH23390 $BP_{\mathrm{ND}}$ was associated with low YGTSS motor score; Fig. 5; Supplementary Table 3). Specifically, a strong anti-correlation was obtained in caudate (*R* = −0.71, *P* = 0.0015) and moderate anti-correlations in putamen (*R* = −0.49, *P* = 0.05) and the combined striatal nuclei (*R* = −0.55, *P* = 0.02). Other tic severity metrics (ATQ and PUTS) did not yield significant correlations (Supplementary Table 4).

### Association between iron and D_1_ receptor availability

**Figure 6:**
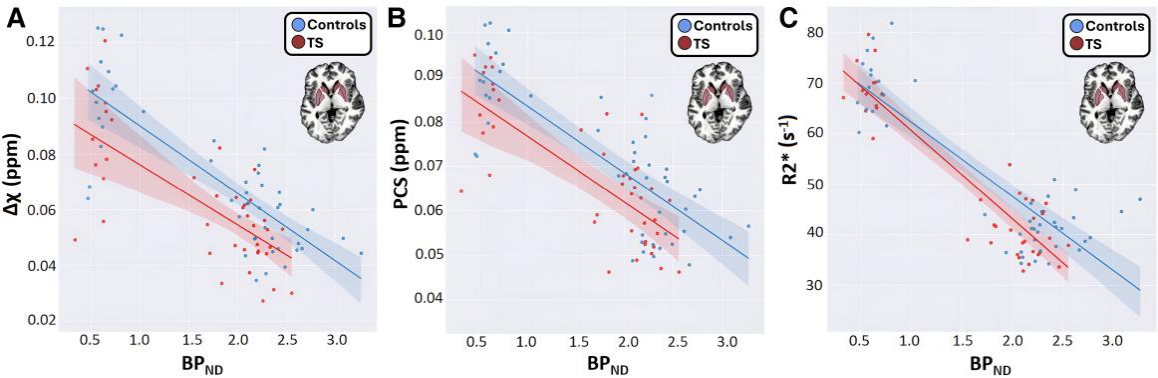
**Correlations between MRI-derived brain iron surrogates and D_1_ receptor availability.** Shown are mean values across bilateral ROIs in the pallidum, caudate and putamen of (**A**) Δχ, (**B**) PCS and (**C**) $R_{2}^{*}$ with [^11^C]SCH23390 $BP_{\mathrm{ND}}$ in the subgroups of control subjects (*n* = 16; blue symbols) and patients with TS (*n* = 15; red symbols) participating in both the PET and 7T MRI studies. Linear regression, y=ax+b, yielded *a* = −0.024 and −0.022 ppm, *b* = 0.11 and 0.098 ppm for Δχ; *a* = −0.015 and −0.015 ppm, *b* = 0.099 and 0.091 ppm for PCS and *a* = −14.5 and −17.3 s^−1^, *b* = 76.7 and 77.3 s^−1^ for $R_{2}^{*}$ in controls and patients, respectively. Note that the regression lines are clearly separated and downshifted for the patients (red) compared with controls (blue) for all three metrics. This is consistent with the observation of reduced levels of iron surrogates (Fig. 2) and [^11^C]SCH23390 $BP_{\mathrm{ND}}$ (Fig. 4) in sub-cortical areas for patients with TS.

Strong negative correlations were obtained between MRI-derived brain iron surrogates and PET-derived [^11^C]SCH23390 $BP_{\mathrm{ND}}$ in basal ganglia nuclei (i.e. high Δχ and $R_{2}^{*}$ were associated with low [^11^C]SCH23390 $BP_{\mathrm{ND}}$; Fig. 6), with a clear distinction between the regression lines for controls (Δχ: *R* = −0.75, *P* = 1.9×10^–10^; PCS: *R* = −0.77, *P*=1.9×10^–10^ and $R_{2}^{*}$: *R* = −0.86, *P*=2×10^–14^) and for patients (Δχ: *R* = −0.71, *P* = 2.3×10^–7^; PCS: *R* = −0.71, *P*=4.7×10^–7^ and $R_{2}^{*}$: *R* = −0.89, *P* = 4×10^–6^). The fact that the patients’ regression lines appear downshifted (compared with the controls’ results) reflects the simultaneous reductions in sub-cortical iron (Fig. 2) and [^11^C]SCH23390 $BP_{\mathrm{ND}}$ (Fig. 4). The strong correlations were partially driven by clustering due to the substantially lower [^11^C]SCH23390 $BP_{\mathrm{ND}}$ in the pallidum compared with the dorsal striatum (caudate/putamen) in all subjects (see Fig. 4).

Moderate negative correlations were also obtained between different serum iron parameters and [^11^C]SCH23390 $BP_{\mathrm{ND}}$ in multiple ROIs (Fig. 3C and D; Supplementary Fig. 5). Specifically, high serum ferritin levels were associated with low [^11^C]SCH23390 $BP_{\mathrm{ND}}$ in the dorsal striatum (*R* = −0.35, *P*=0.037), putamen (*R* = −0.41, *P*=0.016), caudate (*R* = −0.25, *P*=0.037) and pallidum (*R* = −0.39, *P* = 0.015). High total serum iron levels were associated with low [^11^C]SCH23390 $BP_{\mathrm{ND}}$ in the pallidum (*R* = −0.33, *P* = 0.04) and thalamus (*R* = −0.39, *P* = 0.016), and a high serum transferrin saturation was associated with a low [^11^C]SCH23390 $BP_{\mathrm{ND}}$ in the pallidum (*R* = −0.39, *P* = 0.017) (Supplementary Table 2 and Fig. 5).

## Discussion

In this multi-modal investigation, susceptibility-sensitive 7T MRI was combined with [^11^C]SCH23390 PET to assess potential disturbances in brain iron homeostasis in adult patients with TS and their association with D_1_ receptor availability. Compared with most earlier PET studies of TS, which often reported inconclusive results,^88,89^ our case–control design included a relevant number of subjects, allowing to draw more robust statistical conclusions. Also, our patients were off medication in order to minimize confounding pharmacological factors. The results revealed significant reductions or tendencies towards reductions in D_1_ receptor binding associated with TS, prominently in the putamen, where D_1_ receptors are abundant. Furthermore, all surrogate iron markers (Δχ, PCS and $R_{2}^{*}$) indicated similar behaviour of the local iron content in major sub-cortical nuclei, which are known to be involved in motor and cognitive functions. This latter result corroborates recent work in a separate sample.^16^ We further observed that the striatal iron markers Δχ and PCS correlated with serum ferritin levels and that reductions of brain iron surrogates were accompanied by reduced serum transferrin levels in patients with TS. The current study extends previous research with susceptibility-sensitive MRI through its use of advanced methods, notably (i) MRI at 7T, which is more sensitive to Δχ variations than experiments at 3T and (ii) the integration of multiple MRI measures of brain iron—PCS and $R_{2}^{*}$ in addition to Δχ. This allowed a broader interpretation as the indices rely on different physical mechanisms:^20,90^ More specifically, (i) the average magnetic susceptibility within a voxel, which is captured by the GRE signal phase (and thus by Δχ) and (ii) the width of the susceptibility-induced field distribution, which is captured by the amplitude decay (and thus by $R_{2}^{*}$).

Generally, both myelin and iron content are major contributors to Δχ and $R_{2}^{*}$ relaxation in brain tissue. Throughout the brain, including sub-cortex, diamagnetic myelin leads to another voxel contribution (DCS) with partial cancellation of the contrast from paramagnetic iron (PCS). The separation of different susceptibility sources promises—if successful—higher accuracy and precision of iron quantification from an isolated paramagnetic effect.^67,91-95^ However, these recently developed methods require further validation. Our results from a clinical application of the DECOMPOSE approach^67^ lend support to the assumption that susceptibility differences between patients with TS and controls are more specifically quantified with PCS than with the less-specific Δχ and $R_{2}^{*}$. Furthermore, this strengthens the interpretation that the alterations observed with QSM are indeed driven by the sub-cortical iron content and not due to confounding changes in myelination. This is further corroborated by the lack of significant differences in the corresponding analysis of the DCS in the major sub-cortical nuclei except of an isolated observation in the thalamus (Supplementary Fig. 6).

Remarkably, in the putamen, reductions of all iron surrogates for patients versus controls were insignificant (*P* > 0.05), consistent with previous results.^16^ At the same time, the difference in D_1_ receptor availability between groups was highly significant. The dorsal striatum is primarily composed of GABAergic medium-sized spiny projection neurons (MSNs), expressing D_1_ (direct pathway) or D_2_ (indirect pathway) receptors.^96^ The abundance of direct pathway MSNs in the putamen leads to excellent conditions for PET-based D_1_ receptor imaging and, thus, to a high sensitivity in detecting differences between patients and controls. However, the putamen shows substantial structural heterogeneity, affecting the quantification of neurotransmitter markers^97^ and iron deposition.^98^ Therefore, we cannot exclude the possibility that greater spatial variance has rendered differences in the iron content estimates insignificant. By contrast, the neighbouring caudate, which is smaller and structurally more homogenous,^97^ yielded statistically significant differences in both Δχ and PCS as well as larger effect sizes (Supplementary Table 2). The improved separation of the regression lines of patients and controls for Δχ and PCS, compared with $R_{2}^{*}$, was mainly driven by a relatively small $R_{2}^{*}$ difference in the pallidum between patients and controls. As the pallidum is particularly rich in iron,^84,86^ this may indicate an improved sensitivity of Δχ and PCS in dissociating small susceptibility differences at high iron loading, compared with $R_{2}^{*}$.

### Disturbed iron homeostasis in Tourette syndrome

The brain is believed to represent a ‘privileged’ iron compartment, which—under normal conditions—does not respond to peripheral iron variations.^99^ Therefore, information on brain iron *in vivo* is of fundamental interest for identifying a potential role in the pathophysiology of TS. Consistent with the notion that susceptibility-sensitive MRI provides a more specific biomarker than systemic measures, we observed lower brain iron levels in patients with TS, despite insignificant differences in serum ferritin between patients and controls unless adjusted for dietary habits. Remarkably, reduced serum transferrin levels in our patients further indicate abnormal iron homeostasis. It should be noted that transferrin levels in the brain are believed to be too low for a relevant effect on susceptibility-sensitive MRI, whose paramagnetic contributions are primarily determined by ferritin and—in structures like the SN—neuromelanin.^20^

Various studies have linked iron to development, reporting associations with an increased risk of neuropsychiatric conditions.^21-23^ Specifically, human and animal investigations have revealed that early-life iron deficiency might lead to irreversible long-term abnormalities including cognitive impairments, various motor issues and affective/behavioural impairments (depressive, compulsive and anxiety-like symptoms).^100,101^ Such impairments are also exhibited as parts of the symptomatology in early life and adolescence of patients with TS. Sub-cortical Δχ reductions, indicating reduced local brain iron levels, have further been linked to normative transcriptional profiles suggesting that disruptions in iron regulatory mechanisms are involved in TS pathophysiology.^16^ The same study also reported a trend towards a negative correlation of serum ferritin levels with tic severity, which was, however, not replicated in the current work. Overall, these considerations suggest that early-life disturbances in iron regulatory mechanisms may be involved in the pathophysiology of TS. However, as our study included only adults, it does not provide direct evidence for this hypothesis. Neuroimaging studies in children and adolescents are therefore needed for further evaluation.

### Links between brain iron and dopamine

Iron is a cofactor of tyrosine hydroxylase, the key enzyme of dopamine synthesis, and is required in sufficient supplies for the synthesis of dopamine.^99,102^ Moreover, chemical imaging methods have shown that iron accumulates in dopamine vesicles.^103^ Finally, an excess of cytosolic dopamine can be oxidized by ferric iron to dopamine-*o*-quinone, a precursor for the formation of neuromelanin, which is an effective iron chelator and the main iron compound in dopaminergic neurons.^102^ Taken together, this highlights the tight relationship between iron and dopamine in brain regions where dopamine is abundant. Fittingly, recent longitudinal work by Larsen *et al.*^30^ has established a positive association between the transverse relaxation rate $R_{2}^{\prime }$ attributable to magnetic field in homogeneities across a voxel (i.e. another proxy for brain iron related to $R_{2}^{*}$ through $R_{2}^{*}=R_{2}+R_{2}^{\prime }$, where $R_{2}$ is the irreversible transverse relaxation rate) and [^11^C]dihydrotetrabenazine PET measures of pre-synaptic vesicular dopamine storage. Remarkably, the same work further shows a significant decrease of [^11^C]raclopride PET measures of D_2_/D_3_ binding with age in caudate and putamen but a significant $R_{2}^{\prime }$ increase in the same areas (Fig. 1 in Ref.^30^), which bears a striking similarity to our own observation of a negative association between D_1_ binding and the iron surrogates Δχ, PCS and $R_{2}^{*}$. Taken together, this may suggest that different elements of the dopaminergic system (production, storage, transport and receptors) are differentially affected by abnormal iron homeostasis, which is likely to be further modulated by individual compensatory mechanisms. In summary, it appears that our combined data are related to two aspects of sub-cortical dopaminergic transmission: (i) pre-synaptic dopamine storage, which is linked to cellular iron stores and, therefore, indirectly to susceptibility-sensitive MRI and (ii) available post-synaptic D_1_ receptor levels detected by [^11^C]SCH23390 PET.

With regards to post-synaptic dopaminergic functional elements, Gustavsson *et al.*^31^ reported negative correlations between elevated Δχ and reduced D_1_ receptor availability in the pre-frontal cortex in aging. Consistently, our results suggest an association between MRI-derived iron surrogates and D_1_ receptor binding in the basal ganglia. If higher Δχ values would indicate higher tonic dopamine levels, one might assume that this in turn is associated with downregulation of dopamine receptors. However, the reason for the negative association remains ambiguous, as it is strongly influenced by the inclusion of data from different brain regions with quite different normative expressions of iron, dopamine and dopamine receptors. Nevertheless, all correlations of iron measures and D_1_ receptor availability differentiated patients from controls at the group level, and a comparison of the results from homologous regions is informative.

**Figure 7:**
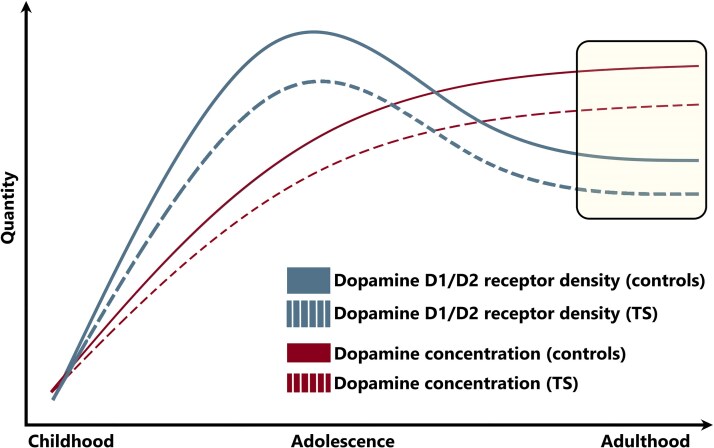
**Schematic depiction of the hypothesized development of pre-synaptic dopamine levels and post-synaptic dopamine receptor expression.** Broken lines show the normal development of the dopaminergic system in the basal ganglia (modified from Fig. 3 of Larsen *et al.*^30^), whereas solid lines show the proposed alterations in TS. The shaded ‘window’ indicates the age range of subjects within the current work (young adults) with reductions in both pre-synaptic dopamine and D_1_ receptor availability.

It is well documented that disruptions in functional elements of the dopaminergic system, which eventually affect tonic/phasic dopaminergic signalling, may occur in combination with iron deficiency.^28^ Decreased D_1_ and D_2_ receptors,^32,104^ and also reduced brain iron, ferritin and transferrin concentrations,^105^ in response to systemic iron deficiency have been documented in rat experiments. Specifically, prolonged iron depletion led to a downregulated density of dopaminergic functional elements (D_1_, D_2_/D_3_) without affecting gene transcription events.^32^ Iron accumulation in the brain is steepest during childhood and adolescence, continuing gradually in adult life.^20,23,106,107^ Rodent data suggest that pre-synaptic dopamine levels increase during development towards adult levels,^108^ consistent with human data indicating a plateau in late adolescence and adulthood.^30,109^ D_1_- and D_2_-like receptors and dopamine transporters were found to gradually decline with age in adult humans,^109^ in line with observations of a steep increase during development and decrease during adult life in rodents.^108^ A qualitative depiction of these transients is presented in Fig. 7. Assuming that patients with TS show similar trends, albeit with reduced pre-synaptic dopamine and also dopamine receptors during development, this could lead to persisting reductions in adulthood as observed here.

Remarkably, our results further indicate a negative correlation of D_1_ receptor availability in the basal ganglia and the severity of motor tics, which links abnormal dopamine transmission to the clinical phenotype. Surprisingly, patients’ motor tic sub-scores were more strongly correlated with [^11^C]SCH23390 $BP_{\mathrm{ND}}$ in the caudate, where the binding only trended towards a reduction in patients (versus controls), whereas the same correlation between tic sub-scores and [^11^C]SCH23390 $BP_{\mathrm{ND}}$ in the putamen was only moderate despite a significant binding reduction in patients. This might suggest that it is the inter-play of different aspects of the dopaminergic system that is involved in the generation of tic. The caudate and putamen are both associated with the expression of D_1_ and D_2_ receptors. Specific sets of striatal neurons can (repeatedly) become abnormally overactive, causing unwanted movements, such as tics.^110,111^ While our results demonstrate abnormalities in D_1_ binding (direct pathway), previous PET studies have indicated a role of abnormalities in the indirect pathway play (D_2_) in TS. Taken together, this fits with the notion that the direct and indirect pathways are intertwined and that communication between their associated medium spiny neurons (MSN) sub-types is crucial for coordinated behaviour and the prevention of inappropriate movements.^112^ Taking together, we hypothesize that chronic iron deficiency during development might contribute to various dopaminergic system abnormalities in TS, including receptor expressions, with more severe abnormality potentially leading to enhanced motor dysregulations.

### Evaluation of hypotheses for Tourette syndrome pathophysiology

Symptoms in TS are thought to be caused by burst-like disinhibition of thalamocortical output, resulting from inappropriate activation of striatal neurons.^110,111^ Increased signal transduction related to dopaminergic dysfunction may be caused at the post-synaptic level by an increased sensitivity to the neurotransmitter due to increased affinity or abundance of dopamine receptors, or, at the pre-synaptic level by abnormally high tonic levels of dopamine or an increased phasic release.^88,89^

*Supersensitive post-synaptic dopamine receptors* were proposed to explain reduced levels of homovanillic acid (HVA) in the CSF of TS patients despite a hyper-dopaminergic system.^113-115^ As HVA represents the final product of dopamine catabolism, it has been used as a biomarker of dopamine turnover in the brain.^116^ However, HVA levels may be confounded by medication, including haloperidol treatment.^115^ Intriguingly, the notion of reduced tonic dopamine would be supported by our finding of reduced striatal iron content, if this serves as a surrogate of pre-synaptic dopamine. Studies in mice have shown that the absence or reduction of dopamine leads to a hyper-sensitivity to dopamine receptor agonists with potential supersensitive behavioural responses.^117,118^ Multiple studies have investigated D_2_-like receptors in TS, albeit with inconsistent results that provide little evidence for an increased number or greater affinity of D_2_-like receptors.^88^ By contrast, a systematic meta-analysis including eight studies rather pointed towards the opposite trend of insignificantly *decreased* striatal D_2_/D_3_ receptor binding.^7^ The supersensitive receptor model further appears to be inconsistent with our result of reduced striatal D_1_ receptor binding in patients not currently receiving medication.

*Dopaminergic hyper-innervation*, that is, an overabundance of striatal dopamine terminals, was suggested to reflect observations of generally increased binding to the dopamine transporter (DAT) and to the vesicular monoamine transporter Type 2, which sequesters cytosolic dopamine into vesicles for storage.^88,89^ The assumption of an increased number of dopaminergic terminals would be consistent with our observation of reduced Δχ, as long as the hypothesis of a positive correlation between striatal dopamine and iron content holds. Alternatively, increased DATs might reflect increased expression per terminal, which would decrease extracellular dopamine.

*Tonic-phasic dysfunction* assumes reduced tonic dopamine levels and a hyper-responsive (spike dependent) phasic dopaminergic system,^89^ although consensus has not been reached.^88^ Low tonic dopamine could be caused by an overactive DAT preventing efficient spillage to the extra-synaptic space and/or altered pre-synaptic dopamine D_2_ auto-receptor binding. A hyper-responsive phasic release could be related to alterations in afferent cortical inputs and/or coexisting DAT abnormality. D_2_ auto-receptor stimulation increases the activity of DATs and inhibits tyrosine hydroxylase and dihydroxyphenylalanine decarboxylase, which would result in decreased dopamine synthesis and packaging into vesicles,^119^ as well as lower tonic dopamine levels. This scenario would be consistent with our hypothesis that reduced striatal iron content correlates with lower levels of pre-synaptic dopamine.

While these considerations point to alterations of intra-synaptic dopamine release, the finding of reduced striatal D_1_ receptor availability that was correlated with motor tic severity contributes to a more comprehensive characterization of TS pathophysiology. It supports the notion of disruptions in multiple functional elements of the dopaminergic system, including the direct pathway. Consistent with this, a recent study *showed that* chemogenetic inhibition of D_1_ or D_2_ receptor–containing nigrostriatal neurons in mice alleviated behavioural patterns of TS.^120^ Coordinated behaviour is thought to result from the integration of output of D_1_ and D_2_ receptor–mediated MSNs,^5,112^ pivoting on the inter-play between the signals in the emergence of tics.^121^ The assumption that excess dopamine leads to an imbalance in the basal ganglia pathways is also supported by fMRI findings of a positive correlation between tic severity and activation of regions involved in the direct pathway.^122^ Stimulation of D_1_ receptors, which are expressed in high levels in striatal MSNs, facilitates glutamate transmission.^123^ Reduced D_1_ receptor availability as observed in the current work might, therefore, be linked to reductions of glutamate levels observed in the basal ganglia of patients with TS.^5^ Involvement of impaired interneurons may also play a critical role.^124^ In a transcriptome analysis, several genes related to striatal GABAergic interneurons showed reduced expression in brain samples from patients with TS, presumably indicating disrupted interneuron signaling.^125^ A recent observation pointing in the same direction was a spatial correlation between reduced Δ*χ* measured in the motor striatum in TS patients and highly enriched terms related to GABAergic signaling in normative gene expression data.^16^

## Conclusion

TS is a multi-faceted neurodevelopmental disorder that encompasses various aspects of dopaminergic disturbances. These may include alterations of intra-synaptic dopamine release and imbalances of the direct and indirect pathway of the basal ganglia. Our study confirms recent findings suggesting that disruptions in iron regulatory mechanisms are also involved in the pathophysiology of TS. Considering that a balance between iron, dopamine and neuromelanin is crucial for cell homeostasis, we hypothesize that disturbances in sub-cortical iron levels are indicative of associated changes in pre-synaptic dopamine levels. Susceptibility-sensitive MRI, which detects local iron content, may therefore provide a surrogate biomarker for sub-cortical dopaminergic transmission. Post-synaptic abnormality in TS is directly demonstrated in the current work by showing associations with reduced D_1_ receptor availability, a finding that has received little attention to date. Remarkably, dopaminergic abnormality correlated with tic severity. Given the inter-play between excitatory, inhibitory and modulatory neurotransmission in the striatum, it is unlikely that the abnormality related to TS is limited to the dopaminergic system. Rather, other neurotransmitter systems are likely involved as well. Dopaminergic abnormalities themselves appear to be associated with disturbances in iron homeostasis.

## Supplementary Material

fcaf104_Supplementary_Data

## Data Availability

The raw data that support the findings of this study are available upon reasonable request from the corresponding author. The data are not publicly available due to data confidentiality agreements protecting the privacy of the research participants. Scripts used for the processing of the raw data are deposited in https://github.com/G-K-O/Iron-Dopamine-GTS_scripts.

## References

[1] Leckman JF . Tourette’s syndrome. Lancet. 2002;360(16):1577–1586. 12443611 10.1016/S0140-6736(02)11526-1

[2] Jakubovski E, Müller-Vahl KR. Gilles de la Tourette syndrome: Symptoms, causes and therapy. Psychother Psychosom Med Psychol. 2017;67(6):252–268. 28722101 10.1055/s-0043-103269

[3] Kwak C, Dat Vuong K, Jankovic J. Premonitory sensory phenomenon in Tourette’s syndrome. Mov Disord. 2003;18(12):1530–1533. 14673893 10.1002/mds.10618

[4] Felling RJ, Singer HS. Neurobiology of Tourette syndrome: Current status and need for further investigation. J Neurosci. 2011;31(35):12387–12395. 21880899 10.1523/JNEUROSCI.0150-11.2011 PMC6703258

[5] Kanaan AS, Gerasch S, García-García I, et al Pathological glutamatergic neurotransmission in Gilles de la Tourette syndrome. Brain. 2017;140(1):218–234. 28007998 10.1093/brain/aww285

[6] Martino D, Leckman JF, eds. Tourette syndrome. 2nd ed. Oxford Academic; 2022.

[7] Hienert M, Gryglewski G, Stamenkovic M, Kasper S, Lanzenberger R. Striatal dopaminergic alterations in Tourette’s syndrome: A meta-analysis based on 16 PET and SPECT neuroimaging studies. Transl Psychiatry. 2018;8(1):143. 30072700 10.1038/s41398-018-0202-y PMC6072751

[8] Draper A, Stephenson MC, Jackson GM, et al Increased GABA contributes to enhanced control over motor excitability in Tourette syndrome. Curr Biol. 2014;24(19):2343–2347. 25264251 10.1016/j.cub.2014.08.038 PMC4188813

[9] Tinaz S, Belluscio BA, Malone P, van der Veen JW, Hallett M, Horovitz SG. Role of the sensorimotor cortex in Tourette syndrome using multimodal imaging. Hum Brain Mapp. 2014;35(12):5834–5846. 25044024 10.1002/hbm.22588 PMC4776755

[10] Müller-Vahl KR, Bindila L, Lutz B, et al Cerebrospinal fluid endocannabinoid levels in Gilles de la Tourette syndrome. Neuropsychopharmacol. 2020;45(8):1323–1329. 10.1038/s41386-020-0671-6 PMC7297729 32272483

[11] Müller-Vahl KR, Fremer C, Beals C, Ivkovic J, Loft H, Schindler C. Endocannabinoid modulation using monoacylglycerol lipase inhibition in Tourette syndrome: A phase 1 randomized, placebo-controlled study. Pharmacopsychiatry. 2022;55(3):148–156. 34847610 10.1055/a-1675-3494 PMC9110099

[12] Pringsheim T, Holler-Managan Y, Okun MS, et al Comprehensive systematic review summary: Treatment of tics in people with Tourette syndrome and chronic tic disorders. Neurology. 2019;92(19):907–915. Erratum: *Neurology* 2019; 93(9): 415. 31061209 10.1212/WNL.0000000000007467 PMC6537130

[13] Gilbert DL, Budman CL, Singer HS, Kurlan R, Chipkin REA. A D1 receptor antagonist, ecopipam, for treatment of tics in Tourette syndrome. Clin Neuropharmacol. 2014;37(1):26–30. 24434529 10.1097/WNF.0000000000000017

[14] Gilbert DL, Murphy TK, Jankovic J, et al Ecopipam, a D1 receptor antagonist, for treatment of Tourette syndrome in children: A randomized, placebo-controlled crossover study. Mov Disord. 2018;33(8):1272–1280. 30192018 10.1002/mds.27457

[15] Gilbert DL, Dubow JS, Cunniff TM, Wanaski SP, Atkinson SD, Mahableshwarkar AR. Ecopipam for Tourette syndrome: A randomized trial. Pediatrics. 2023;151(2):e2022059574. 36628546 10.1542/peds.2022-059574

[16] Kanaan AS, Yu D, Metere R, et al Convergent imaging-transcriptomic evidence for disturbed iron homeostasis in Gilles de la Tourette syndrome. Neurobiol Dis. 2023;185:106252. 37536382 10.1016/j.nbd.2023.106252

[17] Peterson BS, Gore JC, Riddle MA, Cohen DJ, Leckman JF. Abnormal magnetic resonance imaging T_2_ relaxation time asymmetries in Tourette’s syndrome. Psychiatry Res Neuroimaging. 1994;55(4):205–221. 10.1016/0165-1781(95)91246-A 7701035

[18] Gorman DA, Zhu H, Anderson GM, Davies M, Peterson BS. Ferritin levels and their association with regional brain volumes in Tourette’s syndrome. Am J Psychiatry. 2006;163(7):1264–1272. 16816233 10.1176/appi.ajp.163.7.1264 PMC2367153

[19] Ghosh D, Burkman E. Relationship of serum ferritin level and tic severity in children with Tourette syndrome. Childs Nerv Syst. 2017;33(8):1373–1378. 28470381 10.1007/s00381-017-3424-z

[20] Möller HE, Bossoni L, Connor JR, et al Iron, myelin, and the brain: Neuroimaging meets neurobiology. Trends Neurosci. 2019;42(6):384–401. 31047721 10.1016/j.tins.2019.03.009

[21] Cortese S, Lecendreux M, Bernardina BD, Mouren MC, Sbarbati A, Konofal E. Attention-deficit/hyperactivity disorder, Tourette’s syndrome, and restless legs syndrome: The iron hypothesis. Med Hypotheses. 2008;70(6):1128–1132. 18164140 10.1016/j.mehy.2007.10.013

[22] Chen M-H, Su T-P, Chen Y-S, et al Association between psychiatric disorders and iron deficiency anemia among children and adolescents: A nationwide population-based study. BMC Psychiatry. 2013;13(1):161. 23735056 10.1186/1471-244X-13-161 PMC3680022

[23] Degremont A, Jain R, Philippou E, Latunde-Dada GO. Brain iron concentrations in the pathophysiology of children with attention deficit/hyperactivity disorder: A systematic review. Nutr Rev. 2021;79(5):615–626. 32974643 10.1093/nutrit/nuaa065

[24] Crichton R . Iron metabolism: From molecular mechanisms to clinical consequences. 4th ed. Wiley; 2016.

[25] Hare DJ, Double KL. Iron and dopamine: A toxic couple. Brain. 2016;139(4):1026–1035. 26962053 10.1093/brain/aww022

[26] Ferreira A, Neves P, Gozzelino R. Multilevel impacts of iron in the brain: The cross talk between neurophysiological mechanisms, cognition, and social behavior. Pharmaceuticals. 2019;12(3):126. 31470556 10.3390/ph12030126 PMC6789770

[27] Bianco L, Unger E, Beard J. Iron deficiency and neuropharmacology. In: Yehuda S, Mostofsky D, eds. Iron deficiency and overload. Humana Press; 2010:141–158.

[28] Lozoff B . Early iron deficiency has brain and behavior effects consistent with dopaminergic dysfunction. J Nutr. 2011;141(4):740S–746S. 21346104 10.3945/jn.110.131169 PMC3056585

[29] Klein MO, Battagello DS, Cardoso AR, Hauser DN, Bittencourt JC, Correa RG. Dopamine: Functions, signaling, and association with neurological diseases. Cell Mol Neurobiol. 2019;39(1):31–59. 30446950 10.1007/s10571-018-0632-3 PMC11469830

[30] Larsen B, Olafsson V, Calabro F, et al Maturation of the human striatal dopamine system revealed by PET and quantitative MRI. Nat Commun. 2020;11(1):846. 32051403 10.1038/s41467-020-14693-3 PMC7015913

[31] Gustavsson J, Johansson J, Falahati F, et al The iron-dopamine D1 coupling modulates neural signatures of working memory across adult lifespan. Neuroimage. 2023;279:120323. 37582419 10.1016/j.neuroimage.2023.120323

[32] Erikson KM, Jones BC, Hess EJ, Zhang Q, Beard JL. Iron deficiency decreases dopamine D_1_ and D_2_ receptors in rat brain. Pharmacol Biochem Behav. 2001;69(3–4):409–418. 11509198 10.1016/s0091-3057(01)00563-9

[33] Singer HS, Hahn I-H, Moran TH. Abnormal dopamine uptake sites in postmortem striatum from patients with Tourette’s syndrome. Ann Neurol. 1991;30(4):558–562. 1838678 10.1002/ana.410300408

[34] Yoon DY, Gause CD, Leckman JF, Singer HS. Frontal dopaminergic abnormality in Tourette syndrome: A postmortem analysis. J Neurol Sci. 2007;255(1–2):50–56. 17337006 10.1016/j.jns.2007.01.069

[35] Leckman JF, Riddle MA, Hardin MT, et al The Yale Global Tic Severity Scale: Initial testing of a clinician-rated scale of tic severity. J Am Acad Child Adolesc Psychiatry. 1989;28(4):566–573. 2768151 10.1097/00004583-198907000-00015

[36] Abramovitch A, Reese H, Woods DW, et al Psychometric properties of a self-report instrument for the assessment of tic severity in adults with tic disorders. Behav Ther. 2015;46(6):786–796. 26520221 10.1016/j.beth.2015.06.002 PMC5716633

[37] Woods DW, Piacentini J, Himle MB, Chang S. Premonitory Urge for Tics Scale (PUTS): Initial psychometric results and examination of the premonitory urge phenomenon in youths with Tic disorders. J Dev Behav Pediatr. 2005;26(6):397–403. 16344654 10.1097/00004703-200512000-00001

[38] Guy W . ECDEU assessment manual for psychopharmacology–revised (DHEW publ. No. ADM 76-338). US Department of Health, Education, and Welfare, Public Health Service, Alcohol, Drug Abuse, and Mental Health Administration, National Institute of Mental Health, Psychopharmacology Research Branch, Division of Extramural Research Programs; 1976:218–222.

[39] Cavanna EA, Schrag A, Morley D, et al The Gilles de la Tourette syndrome-quality of life scale (GTS-QOL): Development and validation. Neurology. 2008;71(18):1410–1416. 18955683 10.1212/01.wnl.0000327890.02893.61

[40] Buysse DJ, Reynolds CF 3rd, Monk TH, Berman SR, Kupfer DJ. The Pittsburgh sleep quality index: A new instrument for psychiatric practice and research. Psychiatry Res. 1989;28(2):193–213. 2748771 10.1016/0165-1781(89)90047-4

[41] Rösler M, Retz W, Retz-Junginger P, et al Instrumente zur Diagnostik der Aufmerksamkeitsdefizit-/Hyperaktivitätsstörung (ADHS) im Erwachsenenalter. Selbstbeurteilungsskala (ADHS-SB) und Diagnosecheckliste (ADHS-DC). Nervenarzt. 2004;75(9):888–895. 15378249 10.1007/s00115-003-1622-2

[42] Goodman WK, Price LH, Rasmussen SA, et al The Yale-Brown Obsessive-Compulsive Scale. I. Development, use, and reliability. Arch Gen Psychiatry. 1989;47(11):1006–1011. 10.1001/archpsyc.1989.01810110048007 2684084

[43] Foa EB, Huppert JD, Leiberg S, et al The obsessive-compulsive inventory: Development and validation of a short version. Psychol Assess. 2002;14(4):485–496. 12501574

[44] Müller-Vahl KR, Kayser L, Pisarenko A, et al The rage attack questionnaire-revised (RAQ-R): Assessing rage attacks in adults with Tourette syndrome. Front Psychiatry. 2020;10:956. 32063867 10.3389/fpsyt.2019.00956 PMC6997809

[45] Palm L, Haas M, Pisarenko A, Jakubovski E, Müller-Vahl KR. Validation of the rage attack questionnaire-revised (RAQ-R) in a mixed psychiatric population. Front Psychiatry. 2021;12:724802. 34531770 10.3389/fpsyt.2021.724802 PMC8439255

[46] Beck AT, Brown G, Epstein N, Steer RA. An inventory for measuring clinical anxiety: Psychometric properties. J Consult Clin Psychol. 1988;56(6):893–897. 3204199 10.1037//0022-006x.56.6.893

[47] Phan T, Carter O, Adams C, et al Discriminant validity of the hospital anxiety and depression scale, beck depression inventory (II) and Beck anxiety inventory to confirmed clinical diagnosis of depression and anxiety in patients with chronic obstructive pulmonary disease. Chron Respir Dis. 2016;13(3):220–228. 26944070 10.1177/1479972316634604 PMC5720182

[48] Beck AT, Steer RA, Brown GK. BDI-II, Beck depression inventory: Manual. Psychological Corp.; 1996.

[49] Kühner C, Bürger C, Keller F, Hautzinger M. Reliabilität und Validität des revidierten Beck-Depressionsinventars (BDI-II). Befunde aus deutschsprachigen Stichproben. Nervenarzt. 2007;78(6):651–656. 16832698 10.1007/s00115-006-2098-7

[50] Baron-Cohen S, Wheelwright S, Skinner R, Martin J, Clubley E. The autism-spectrum quotient (AQ): Evidence from Asperger syndrome/high-functioning autism, males and females, scientists and mathematicians. J Autism Dev Disord. 2001;31(1):5–17. 11439754 10.1023/a:1005653411471

[51] Marques JP, Kober T, Krueger G, et al MP2RAGE, a self bias-field corrected sequence for improved segmentation and T_1_-mapping at high field. Neuroimage. 2010;49(2):1271–1281. 19819338 10.1016/j.neuroimage.2009.10.002

[52] Griswold MA, Jakob PM, Heidemann RM, et al Generalized autocalibrating partially parallel acquisitions (GRAPPA). Magn Reson Med. 2002;47(6):1202–1210. 12111967 10.1002/mrm.10171

[53] Feinberg DA, Hale JD, Watts JC, Kaufman L, Mark A. Halving MR imaging time by conjugation: Demonstration at 3.5 kG. Radiology. 1986;161(2):527–531. 3763926 10.1148/radiology.161.2.3763926

[54] Frahm J, Haase A, Matthaei D. Rapid three-dimensional MR imaging using the FLASH technique. J Comput Assist Tomogr. 1986;10(2):363–368. 3950172 10.1097/00004728-198603000-00046

[55] Halldin C, Stone-Elander S, Farde L, et al Preparation of ^11^C-labelled SCH 23390 for in vivo study of dopamine D-1 receptors using positron emission tomography. Int J Rad Appl Instrum A. 1986;37(10):1039–1043. 3027000 10.1016/0883-2889(86)90044-4

[56] Kaller S, Rullmann M, Patt M, et al Test-retest measurements of dopamine D_1_-type receptors using simultaneous PET/MRI imaging. Eur J Nucl Med Mol Imaging. 2017;44(6):1025–1032. 28197685 10.1007/s00259-017-3645-0

[57] Chen Y, An H. Attenuation correction of PET/MR imaging. Magn Reson Imaging Clin N Am. 2017;25(2):245–255. 28390526 10.1016/j.mric.2016.12.001 PMC5385843

[58] Schofield MA, Zhu Y. Fast phase unwrapping algorithm for interferometric applications. Opt Lett. 2003;28(14):1194–1196. 12885018 10.1364/ol.28.001194

[59] Li W, Wu B, Liu C. Quantitative susceptibility mapping of human brain reflects spatial variation in tissue composition. NeuroImage. 2011;55(4):1645–1656. 21224002 10.1016/j.neuroimage.2010.11.088 PMC3062654

[60] Jenkinson M, Beckmann CF, Behrens TE, Woolrich MW, Smith SM. FSL. NeuroImage. 2012;62(2):782–790. 21979382 10.1016/j.neuroimage.2011.09.015

[61] Schweser F, Deistung A, Lehr BW, Reichenbach JR. Quantitative imaging of intrinsic magnetic tissue properties using MRI signal phase: An approach to in vivo brain iron metabolism? Neuroimage. 2011;54(4):2789–2807. 21040794 10.1016/j.neuroimage.2010.10.070

[62] Özbay PS, Deistung A, Feng X, Nanz D, Reichenbach JR, Schweser F. A comprehensive numerical analysis of background phase correction with V-SHARP. NMR Biomed. 2017;30(4):e3550. 10.1002/nbm.3550 PMC5136354 27259117

[63] Li W, Wang N, Yu F, et al A method for estimating and removing streaking artifacts in quantitative susceptibility mapping. Neuroimage. 2015;108:111–122. 25536496 10.1016/j.neuroimage.2014.12.043 PMC4406048

[64] Wu B, Li W, Avram AV, Gho S-M, Liu C. Fast and tissue-optimized mapping of magnetic susceptibility and T2* with multi-echo and multi-shot spirals. Neuroimage. 2012;59(1):297–305. 21784162 10.1016/j.neuroimage.2011.07.019 PMC3235505

[65] Straub S, Schneider TM, Emmerich J, et al Suitable reference tissues for quantitative susceptibility mapping of the brain. Magn Reson Med. 2017;78(1):204–214. 27529579 10.1002/mrm.26369

[66] Pei M, Nguyen TD, Thimmappa ND, et al Algorithm for fast monoexponential fitting based on auto-regression on linear operations (ARLO) of data. Magn Reson Med. 2015;73(2):843–850. Erratum: *Magn Reson Med.* 2019;82(4):1576. 24664497 10.1002/mrm.25137 PMC4175304

[67] Chen J, Gong N-J, Chaim KT, García Otaduy MC, Liu C. Decompose quantitative susceptibility mapping (QSM) to sub-voxel diamagnetic and paramagnetic components based on gradient-echo MRI data. Neuroimage. 2021;242:118477. 34403742 10.1016/j.neuroimage.2021.118477 PMC8720043

[68] Byrge L, Kennedy DP. Identifying and characterizing systematic temporally-lagged BOLD artifacts. Neuroimage. 2018;171:376–392. 29288128 10.1016/j.neuroimage.2017.12.082 PMC5857478

[69] Ichise M, Liow J-S, Lu J-Q, et al Linearized reference tissue parametric imaging methods: Application to [^11^C]DASB positron emission tomography studies of the serotonin transporter in human brain. J Cereb Blood Flow Metab. 2003;23(9):1096–1112. 12973026 10.1097/01.WCB.0000085441.37552.CA

[70] Avants BB, Epstein CL, Grossman M, Gee JC. Symmetric diffeomorphic image registration with cross-correlation: Evaluating automated labeling of elderly and neurodegenerative brain. Med Image Anal. 2008;12(1):26–41. 17659998 10.1016/j.media.2007.06.004 PMC2276735

[71] Patenaude B, Smith SM, Kennedy DN, Jenkinson M. A Bayesian model of shape and appearance for subcortical brain segmentation. Neuroimage. 2011;56(3):907–922. 21352927 10.1016/j.neuroimage.2011.02.046 PMC3417233

[72] Iglesias J, Van Leemput K, Bhatt P, et al Bayesian segmentation of brainstem structures in MRI. Neuroimage. 2015;113:184–195. 25776214 10.1016/j.neuroimage.2015.02.065 PMC4434226

[73] Dong P, Guo Y, Gao Y, et al Multi-atlas based segmentation of brainstem nuclei from MR images by deep hyper-graph learning. In: Wu G, Coupé P, Zhan Y, Munsell B, Rueckert D, eds. Patch-based techniques in medical imaging. Patch-MI 2016. Springer; 2016:51–59. 10.1007/978-3-319-47118-1_7 PMC5868975 29594262

[74] Beliveau V, Nørgaard M, Birkl C, Seppi K, Scherfler C. Automated segmentation of deep brain nuclei using convolutional neural networks and susceptibility weighted imaging. Hum Brain Mapp. 2021;42(15):4809–4822. 34322940 10.1002/hbm.25604 PMC8449109

[75] Alkemade A, Mulder MJ, Trutti AC, Forstmann BU. Manual delineation approaches for direct imaging of the subcortex. Brain Struct Funct. 2022;227(1):219–297. 34714408 10.1007/s00429-021-02400-x PMC8741717

[76] Cheng Z, He N, Huang P, et al Imaging the nigrosome 1 in the substantia nigra using susceptibility weighted imaging and quantitative susceptibility mapping: An application to Parkinson’s disease. NeuroImage Clin. 2020;25:102103. 31869769 10.1016/j.nicl.2019.102103 PMC6933220

[77] Brammerloh M, Morawski M, Friedrich I, et al Measuring the iron content of dopaminergic neurons in substantia nigra with MRI relaxometry. Neuroimage. 2021;239:118255. 34119638 10.1016/j.neuroimage.2021.118255 PMC8363938

[78] Morfini F, Whitfield-Gabrieli S, Nieto-Castañón A. Functional connectivity MRI quality control procedures in CONN. Front Neurosci. 2023;17:1092125. 37034165 10.3389/fnins.2023.1092125 PMC10076563

[79] De Keyser J, Claeys A, De Backer J-P, Ebinger G, Roels F, Vauquelin G. Autoradiographic localization of D_1_ and D_2_ dopamine receptors in the human brain. Neurosci Lett. 1988;91(2):142–147. 2972942 10.1016/0304-3940(88)90758-6

[80] Hall H, Sedvall G, Magnusson O, Kopp J, Halldin C, Farde L. Distribution of D_1_- and D_2_-dopamine receptors, and dopamine and its metabolites in the human brain. Neuropsychopharmacology. 1994;11(4):245–256. 7531978 10.1038/sj.npp.1380111

[81] Cadet JL, Jayanthi S, McCoy MT, Beauvais G, Cai NS. Dopamine D1 receptors, regulation of gene expression in the brain, and neurodegeneration. CNS Neurol Disord Drug Targets. 2010;9(5):526–538. 20632973 10.2174/187152710793361496 PMC3803153

[82] Jones-Tabah J, Mohammad H, Paulus EG, Clarke PBS, Hébert TE. The signaling and pharmacology of the dopamine D1 receptor. Front Cell Neurosci. 2022;15:806618. 35110997 10.3389/fncel.2021.806618 PMC8801442

[83] Pawlak R, Berger J, Hines I. Iron status of vegetarian adults: A review of literature. Am J Lifestyle Med. 2016;12(6):486–498. 30783404 10.1177/1559827616682933 PMC6367879

[84] Hallgren B, Sourander P. The effect of age on the non-haemin iron in the human brain. J Neurochem. 1958;3(1):41–51. 13611557 10.1111/j.1471-4159.1958.tb12607.x

[85] Péran P, Cherubini A, Luccichenti G, et al Volume and iron content in basal ganglia and thalamus. Hum Brain Mapp. 2009;30(8):2667–2675. 19172651 10.1002/hbm.20698 PMC6871035

[86] Langkammer C, Schweser F, Krebs N, et al Quantitative susceptibility mapping (QSM) as a means to measure brain iron? A post mortem validation study. Neuroimage. 2012;62(3):1593–1599. 22634862 10.1016/j.neuroimage.2012.05.049 PMC3413885

[87] Murakami Y, Kakeda S, Watanabe K, et al Usefulness of quantitative susceptibility mapping for the diagnosis of Parkinson disease. AJNR Am J Neuroradiol. 2015;36(6):1102–1108. 25767187 10.3174/ajnr.A4260 PMC8013031

[88] Maia TV, Conceição VA. Dopaminergic disturbances in Tourette syndrome: An integrative account. Biol Psychiatry. 2018;84(5):332–344. 29656800 10.1016/j.biopsych.2018.02.1172

[89] Singer HS . Tourette syndrome: Circuits and neurotransmitters. In: Martino D, Leckman JF, eds. Tourette syndrome. 2nd ed. Oxford Academic; 2022:231–256.

[90] Duyn JH . Studying brain microstructure with magnetic susceptibility contrast at high-field. Neuroimage. 2018;168:152–161. 28242317 10.1016/j.neuroimage.2017.02.046 PMC5569005

[91] Emmerich J, Bachert P, Ladd ME, Straub S. On the separation of susceptibility sources in quantitative susceptibility mapping: Theory and phantom validation with an in vivo application to multiple sclerosis lesions of different age. J Magn Reson. 2021;330:107033. 34303117 10.1016/j.jmr.2021.107033

[92] Shin H-G, Lee J, Yun YH, et al χ-separation: Magnetic susceptibility source separation toward iron and myelin mapping in the brain. Neuroimage. 2021;240:118371. 34242783 10.1016/j.neuroimage.2021.118371

[93] Dimov AV, Nguyen TD, Gillen KM, et al Susceptibility source separation from gradient echo data using magnitude decay modeling. J Neuroimaging. 2022;32(5):852–859. 35668022 10.1111/jon.13014

[94] Zhang Z, Cho Z, Wang L, et al Blip up-down acquisition for spin- and gradient-echo imaging (BUDA-SAGE) with self-supervised denoising enables efficient T_2_, T_2_*, para- and dia-magnetic susceptibilty mapping. Magn Reson Med. 2022;88(2):633–650. 35436357 10.1002/mrm.29219

[95] Li Z, Feng R, Liu Q, et al APART-QSM: An improved sub-voxel quantitative susceptibility mapping for susceptibility source separation using an iterative data fitting method. Neuroimage. 2023;274:120148. 37127191 10.1016/j.neuroimage.2023.120148

[96] Smith Y, Bevan MD, Shink E, Bolam JP. Microcircuity of the direct and indirect pathways of the basal ganglia. Neuroscience. 1998;86(2):353–387. 9881853 10.1016/s0306-4522(98)00004-9

[97] Hörtnagl H, Pifl C, Hörtnagl E, Reiner A, Sperk G. Distinct gradients of various neurotransmitter markers in caudate nucleus and putamen of the human brain. J Neurochem. 2020;152(6):650–662. 31608979 10.1111/jnc.14897 PMC7078952

[98] Krishnamurthy B, Haacke EM, Ayaz M, Harder S, Kid D, Kirsch W. Iron deposition in the putamen. In: *Proceedings of the 14th Annual Meeting of the ISMRM*. 2006:2656.

[99] Ward RJ, Zucca FA, Duyn JH, Crichton RR, Zecca L. The role of iron in brain ageing and neurodegenerative disorders. Lancet Neurol. 2014;13(10):1045–1060. 25231526 10.1016/S1474-4422(14)70117-6 PMC5672917

[100] Felt BT, Beard JL, Schallert T, et al Persistent neurochemical and behavioral abnormalities in adulthood despite early iron supplementation for perinatal iron deficiency anemia in rats. Behav Brain Res. 2006;171(2):261–270. 16713640 10.1016/j.bbr.2006.04.001 PMC1851886

[101] Kennedy BC, Wallin DJ, Tran PV, Georgieff MK. Long-term brain and behavioral consequences of early-life iron deficiency. In: Reissland N, Kisilevsky B, eds. Fetal development. Springer; 2016:295–316.

[102] Zucca FA, Segura-Aguilar J, Ferrari E, et al Interactions of iron, dopamine and neuromelanin pathways in brain aging and Parkinson’s disease. Prog Neurobiol. 2017;155:96–119. 26455458 10.1016/j.pneurobio.2015.09.012 PMC4826627

[103] Ortega R, Cloetens P, Devès G, Carmora A, Bohic S. Iron storage within dopamine neurovesicles revealed by chemical nano-imaging. PLoS One. 2007;2(9):e925. 17895967 10.1371/journal.pone.0000925 PMC1976597

[104] Ben-Shachar D, Ashkenazi R, Youdim MBH. Long-term consequence of early iron-deficiency on dopaminergic neurotransmission in rats. Int J Dev Neurosci. 1986;4(1):81–88. 2844061 10.1016/0736-5748(86)90019-5

[105] Chen Q, Connor JR, Beard JL. Brain iron, transferrin and ferritin concentrations are altered in developing iron-deficient rats. J Nutr. 1995;125(6):1529–1535. 7782907 10.1093/jn/125.6.1529

[106] Zecca L, Youdim MB, Riederer P, Connor JR, Crichton RR. Iron, brain ageing and neurodegenerative disorders. Nat Rev Neurosci. 2004;5(11):863–873. 15496864 10.1038/nrn1537

[107] Treit S, Naji N, Seres P, et al R2* and quantitative susceptibility mapping in deep gray matter of 498 healthy controls from 5 to 90 years. Hum Brain Mapp. 2021;42(14):4597–4610. 34184808 10.1002/hbm.25569 PMC8410539

[108] Giorgi O, De Montis G, Porceddu ML, et al Developmental and age-related changes in D1-dopamine receptors and dopamine content in the rat striatum. Brain Res. 1987;432(2):283–290. 2960426 10.1016/0165-3806(87)90053-8

[109] Karrer TM, Josef AK, Mata R, Morris ED, Samanez-Larkin GR. Reduced dopamine receptors and transporters but not synthesis capacity in normal aging adults: A meta-analysis. Neurobiol Aging. 2017;57:36–46. 28599217 10.1016/j.neurobiolaging.2017.05.006 PMC5645072

[110] Mink JW . Basal ganglia dysfunction in Tourette’s syndrome: A new hypothesis. Pediatr Neurol. 2001;25(3):190–198. 11587872 10.1016/s0887-8994(01)00262-4

[111] Albin RL, Mink JW. Recent advances in Tourette syndrome research. Trends Neurosci. 2006;29(3):175–182. 16430974 10.1016/j.tins.2006.01.001

[112] Calabresi P, Picconi B, Tozzi A, Ghiglieri V, Di Filippo M. Direct and indirect pathways of basal ganglia: A critical reappraisal. Nat Neurosci. 2014;17(8):1022–1030. 25065439 10.1038/nn.3743

[113] Cohen DJ, Shaywitz BA, Caparulo B, Young JG, Bowers MB Jr. Chronic, multiple tics of Gilles de la Tourette’s disease. CSF acid monoamine metabolites after probenecid administration. Arch Gen Psychiatry. 1978;35(2):245–250. 272137 10.1001/archpsyc.1978.01770260123015

[114] Butler IJ, Koslow SH, Seifert WE Jr, Caprioli RM, Singer HS. Biogenic amine metabolism in Tourette syndrome. Ann Neurol. 1979;6(1):37–39. 292354 10.1002/ana.410060109

[115] Singer HS, Butler IJ, Tune LE, Seifert WE Jr, Coyle JT. Dopaminergic dysfunction in Tourette syndrome. Ann Neurol. 1982;12(4):361–366. 6184010 10.1002/ana.410120408

[116] Marín-Valencia I, Serrano M, Ormazabal A, et al Biochemical diagnosis of dopaminergic disturbances in paediatric patients: Analysis of cerebrospinal fluid homovanillic acid and other biogenic amines. Clin Biochem. 2008;41(16–17):1306–1315. 18790694 10.1016/j.clinbiochem.2008.08.077

[117] Riffee WH, Wilcox RE, Vaughn DM, Smith RV. Dopamine receptor sensitivity after chronic dopamine agonists. Psychopharmacology (Berl). 1982;77(2):146–149. 6812131 10.1007/BF00431937

[118] Kim DS, Szczypka MS, Palmiter RD. Dopamine-deficient mice are hypersensitive to dopamine receptor agonists. J Neurosci. 2000;20(12):4405–4413. 10844009 10.1523/JNEUROSCI.20-12-04405.2000 PMC6772455

[119] Ford CP . The role of D2-autoreceptors in regulating dopamine neuron activity and transmission. Neuroscience. 2014;282:13–22. 24463000 10.1016/j.neuroscience.2014.01.025 PMC4108583

[120] Lin L, Lan Y, Zhu H, et al Effects of chemogenetic inhibition of D_1_ or D_2_ receptor-containing neurons of the substantia nigra and striatum in mice with Tourette syndrome. Front Mol Neurosci. 2021;14:779436. 34955745 10.3389/fnmol.2021.779436 PMC8696039

[121] Caligiore D, Mannella F, Arbib MA, Baldassarre G. Dysfunctions of the basal ganglia-cerebellar-thalamo-cortical system produce motor tics in Tourette syndrome. PLoS Comput Biol. 2017;13(3):e1005395. 28358814 10.1371/journal.pcbi.1005395 PMC5373520

[122] Baym CL, Corbett BA, Wright SB, Bunge SA. Neural correlates of tic severity and cognitive control in children with Tourette syndrome. Brain. 2008;131(1):165–179. 18056159 10.1093/brain/awm278

[123] Surmeier DJ, Ding J, Day M, Wang Z, Shen W. D1 and D2 dopamine-receptor modulation of striatal glutamatergic signaling in striatal medium spiny neurons. Trends Neurosci. 2007;30(5):228–235. 17408758 10.1016/j.tins.2007.03.008

[124] Dwyer JB . A developmental perspective of dopaminergic dysfunction in Tourette syndrome. Biol Psychiatry. 2018;84(5):e33–e35. 30115244 10.1016/j.biopsych.2018.07.008

[125] Lennington JB, Coppola C, Kataoka-Sasaki Y, et al Transcriptome analysis of the human striatum in Tourette syndrome. Biol Psychiatry. 2016;79(5):372–382. 25199956 10.1016/j.biopsych.2014.07.018 PMC4305353

