# Supplementary material for: Abnormalities of iron homeostasis and the dopaminergic system in Tourette syndrome revealed by 7T MRI and PET
Source: Brain Commun. 2025 Mar 10;7(2):fcaf104. doi: 10.1093/braincomms/fcaf104 (PMC11961303; doi:10.1093/braincomms/fcaf104)
Supplement: fcaf104_Supplementary_Data [file fcaf104_supplementary_data.docx]

**—SUPPLEMENTARY MATERIAL—**

**Abnormalities of iron homeostasis and the dopaminergic system in Tourette syndrome revealed by 7T MRI and PET**

Dimitrios G. Gkotsoulias^1^, Michael Rullmann^2^, Simon Schmitt^3^, Anna Bujanow^1^, Franziska Zientek^2^, Konstantin Messerschmidt^2^, André Pampel^1^, Amira-Philine Büttner^1^, Andreas Schildan^2^, Osama Sabri^2^, Kirsten Müller-Vahl^3,^*, Henryk Barthel^2,^*, Harald E. Möller^1,4,^*

*^1^ Max Planck Institute for Human Cognitive and Brain Sciences, Leipzig, Germany*

*^2^ Department of Nuclear Medicine, Leipzig University Medical Center, Leipzig, Germany*

*^3^ Department of Psychiatry, Social Psychiatry and Psychotherapy, Hannover Medical School, Hannover, Germany*

*^4^ Felix Bloch Institute for Solid State Physics, Leipzig University, Leipzig, Germany*

*^*^ Harald E. Möller, Kirsten Müller-Vahl and Henryk Barthel contributed equally to this work.*

**Abbreviations**

ADHD = attention deficit/hyperactivity disorder; ANTs = Advanced Normalization Tools; AQ-k = Autismus-Spektrum-Quotient, Kurzversion; ARLO = Auto-Regression on Linear Operations; ATQ = Adult Tic Questionnaire; BAI = Beck Anxiety Inventory; BDI-II = Beck Depression Inventory, 2^nd^ revision; CAARS = Conners’ Adult ADHD Rating Scale; CI = confidence interval; CGI–S; Clinical Global Impressions–Severity; [^11^C]SCH23390 = [^11^C](*R*)-2,3,4,5-tetrahydro-8-chloro-3-methyl-5-phenyl-1H-3-benzazepine-7-ol; CSF = cerebrospinal fluid; D_1_, D_2_, D_3_ = dopamine receptor 1, 2, 3; DAT = dopamine transporter; DCS = diamagnetic contribution to susceptibility; DECOMPOSE = DiamagnEtic COMponent and Paramagnetic cOmponent Separation; DOPA = dihydroxyphenylalanine; DSM-IV = Diagnostic and Statistical Manual of Mental Disorders, 4^th^ edition; FDR = false discovery rate; fMRI = functional magnetic resonance imaging; FOV = field of view; Ft = ferritin, FSL = FMRIB Software Library; GABA = γ-aminobutyric acid; GRAPPA = GeneRalized Autocalibrating Partially Parallel Acquisitions; GRE = Gradient-Recalled Echo; GTS–QOL; Gilles de la Tourette Syndrome–Quality Of Life; HVA = homovanillic acid; ME = multi echo; MP2RAGE = Magnetization Prepared 2 Rapid Acquisition Gradient Echoes; MR = magnetic resonance; MRI = magnetic resonance imaging; MRTM2 = Multilinear Reference Tissue Model 2; MSN = medium-sized spiny neuron; OCB = obsessive compulsive behavior; OCD = obsessive compulsive disorder; PCS = paramagnetic contribution to susceptibility; PET = positron emission tomography; PSQI = Pittsburgh Sleep Quality Index; PUTS = Premonitory Urge for Tics Scale; QSM = quantitative susceptibility mapping; RAQ = Rage Attack Questionnaire; RN = red nucleus; ROI = region of interest; SN = substantia nigra; SNR = signal-to-noise ratio; SPM12 = statistical parametric mapping, revision 12; StN = subthalamic nucleus; Tf = transferrin; TS = Tourette syndrome; VMAT2 = vesicular monoamine transporter type 2; V-SHARP = Variable-kernel Sophisticated Harmonic Artifact Reduction for Phase data; WM = white matter; Y-BOCS; Yale-Brown Obsessive Compulsive Scale; YGTSS = Yale Global Tic Severity Scale.

**Mathematical symbols**

| $\text{BP}_{\text{ND}}$: non-displaceable binding potential;  BW: bandwidth;  $d$: Cohen’s $d$;  $f_{p}$: partial-Fourier factor;  $n$: integer number;  $P$: error probability;  $P_{FDR}$: $P$-value after FDR correction;  $R$: Pearson’s or Spearman’s correlation coefficient;  $R_{2}$: irreversible transverse relaxation rate  $R_{2}$: effective transverse relaxation rate; | $R_{2}^{'}$: transverse relaxation rate attributable to magnetic field inhomogeneities;  $T_{1}$: longitudinal relaxation time;  $T_{2}$: effective transverse relaxation time;  TA: acquisition time;  TE: echo time;  TI: inversion time;  TR: repetition time;  $t$: *t*-value;  $\alpha$: RF pulse flip angle;  $\Delta\chi$: bulk magnetic susceptibility. |
| --- | --- |

**Supplementary Table 1. Statistical group comparison of MRI-derived brain iron surrogate metrics and PET-derived [^11^C]SCH23390** $\text{BP}_{\text{ND}}$**.** Results (mean values ± one standard deviation and confidence intervals) are shown for preselected subcortical nuclei. *P*_FDR_ in bold font indicates statistical significance after FDR-correction. Cohen’s $d$ in bold font indicates medium/large effect size ($d$>0.5).

| **ROI** | **Controls Mean ± SD [*CI*]** | **TS Mean ± SD [*CI*]** | ***t*-val.** | ***P* uncorr.** | $\text{P}_{\text{FDR}}$ | **Cohen’s *d*** |
| --- | --- | --- | --- | --- | --- | --- |
| $\Delta\chi$ ***[ppm]*** | | | | | | |
| **SN** | 0.079 ± 0.012 [0.075–0.083] | 0.068 ± 0.013 [0.063–0.073] | –3.282 | **0.001** | **0.003** | **0.90** |
| **StN** | 0.067 ± 0.015 [0.062–0.072] | 0.060 ± 0.012 [0.055–0.065] | –1.820 | **0.037** | **0.042** | 0.46 |
| **RN** | 0.065 ± 0.018 [0.059–0.071] | 0.052 ± 0.012 [0.046–0.056] | –3.044 | **0.001** | **0.003** | **0.76** |
| **Striatum** | 0.059 ± 0.006 [0.057–0.061] | 0.054 ± 0.008 [0.050–0.057] | –2.396 | **0.010** | **0.013** | 0.21 |
| **Putamen** | 0.049 ± 0.007 [0.047–0.051] | 0.046 ± 0.011 [0.041–0.050] | –1.494 | 0.072 | 0.072 | 0.43 |
| **Caudate** | 0.069 ± 0.009 [0.066–0.072] | 0.062 ± 0.010 [0.057–0.066] | –2.403 | **0.010** | **0.013** | **0.66** |
| **Pallidum** | 0.104 ± 0.017 [0.098–0.110] | 0.089 ± 0.015 [0.083–0.095] | –3.162 | **0.001** | **0.002** | **1.01** |
| **Thalamus** | 0.007 ± 0.005 [0.053–0.086] | 0.004 ± 0.005 [0.002–0.006] | –2.470 | **0.008** | **0.013** | **0.68** |
| ***PCS [ppm]*** | | | | | | |
| **SN** | 0.079 ± 0.007 [0.077–0.081] | 0.072 ± 0.008 [0.068–0.075] | –3.380 | **0.0008** | **0.002** | **0.84** |
| **StN** | 0.074 ± 0.009 [0.071–0.077] | 0.068 ± 0.007 [0.065–0.071] | –2.090 | **0.020** | **0.040** | 0.43 |
| **RN** | 0.072 ± 0.009 [0.069–0.075] | 0.065 ± 0.007 [0.062–0.068] | –3.060 | **0.001** | **0.002** | **0.80** |
| **Striatum** | 0.065 ± 0.006 [0.063–0.067] | 0.062 ± 0.007 [0.059–0.065] | –1.787 | **0.041** | 0.051 | **0.51** |
| **Putamen** | 0.056 ± 0.007 [0.054–0.058] | 0.053 ± 0.007 [0.050–0.056] | –1.200 | 0.110 | 0.110 | 0.34 |
| **Caudate** | 0.074 ± 0.008 [0.071–0.077] | 0.070 ± 0.009 [0.066–0.074] | –1.240 | **0.028** | **0.045** | **0.53** |
| **Pallidum** | 0.092 ± 0.009 [0.089–0.095] | 0.081 ± 0.012 [0.076–0.086] | –3.340 | **0.001** | **0.002** | **0.98** |
| **Thalamus** | 0.033 ± 0.003 [0.032–0.034] | 0.031 ± 0.003 [0.029–0.032] | –1.700 | **0.045** | 0.051 | 0.44 |
| $R_{2}$ ***[s^–1^]*** | | | | | | |
| **SN** | 61.0 ± 4.7 [59.4–62.6] | 56.0 ± 6.4 [53.3–58.7] | –1.870 | **0.003** | **0.024** | 0.09 |
| **StN** | 59.8 ± 5.1[58.1–61.5] | 56.2 ± 6.2 [53.6–58.8] | –1.740 | **0.040** | 0.100 | 0.49 |
| **RN** | 56.5 ± 5.9 [54.5–58.5] | 53.8 ± 5.7 [51.4–56.2] | –1.630 | **0.050** | 0.100 | 0.31 |
| **Striatum** | 42.2 ± 3.6 [41.0–43.4] | 41.8 ± 3.9 [40.2–43.4] | -0.322 | 0.374 | 0.434 | 0.09 |
| **Putamen** | 45.3 ± 4.6[43.8–46.8] | 44.9 ± 4.9 [42.9–46.9] | –0.290 | 0.380 | 0.434 | 0.08 |
| **Caudate** | 39.4 ± 3.2 [38.3–40.5] | 38.7 ± 3.6 [37.2–40.2] | –0.300 | 0.380 | 0.434 | 0.09 |
| **Pallidum** | 72.0 ± 5.6 [70.1–73.9] | 69.3 ± 4.8 [67.3–71.3] | –1.870 | **0.030** | 0.100 | 0.49 |
| **Thalamus** | 36.2 ± 1.8 [35.6–36.8] | 36.6 ± 1.6 [35.9–37.3] | –0.840 | 0.790 | 0.790 | 0.12 |
| ***[^11^C]SCH23390*** $\text{BP}_{\text{ND}}$ | | | | | | |
| **Striatum** | 2.31 ± 0.29 [2.19–2.44] | 2.07 ± 0.21 [1.97–2.17] | –2.874 | **0.006** | **0.015** | **0.90** |
| **Putamen** | 2.39 ± 0.25 [2.28–2.50] | 2.13 ± 0.23 [2.02–2.23] | –3.252 | **0.002** | **0.010** | **1.07** |
| **Caudate** | 2.19 ± 0.28 [2.07–2.31] | 2.01 ± 0.23 [1.91–2.12] | –2.08 | **0.044** | 0.060 | **0.65** |
| **Pallidum** | 0.63 ± 0.12 [0.58–0.68] | 0.58 ± 0.10 [0.54–0.63] | –1.246 | 0.22 | 0.250 | 0.44 |
| **Thalamus** | 0.37 ± 0.06 [0.35–0.40] | 0.35 ± 0.04 [0.33–0.36] | –1.706 | 0.097 | 0.112 | **0.53** |

**CI** = 95% confidence interval; ***P*_FDR_** = *P*-values after FDR correction; ***P* uncorr.** = *P*-values before FDR correction; **RN** = red nucleus; **SD**: standard deviation; **SN** = substantia nigra; **StN** = subthalamic nucleus; **striatum** = combined putamen and caudate.

**Supplementary Table 2. Correlations of neuroimaging parameters and serum measures of iron status.** Pearson’s coefficients $R$ (roman font) are presented above uncorrected $P$-values (italic font) for correlations between the serum measures total iron, ferritin, transferrin, transferrin saturation and soluble transferrin receptors and the MRI parameters Δχ, PCS and $R_{2}$ as well as the PET-derived [^11^C]SCH23390 $\text{BP}_{\text{ND}}$ in different subcortical ROIs. The cells marked with grey shading denote significant correlations ($R$>0.2 and $P$<0.05).

|  | **SN** | **StN** | **RN** | **Striatum** | **Putamen** | **Caudate** | **Pallidum** | **Thalamus** |
| --- | --- | --- | --- | --- | --- | --- | --- | --- |
| ***Correlations with Δχ*** | | | | | | | | |
| **Iron** | 0.13 *0.34* | 0.003   *0.98* | –0.065 *0.65* | 0.083 *0.55* | –0.032 *0.82* | 0.15 *0.29* | 0.02 *0.89* | 0.026 *0.85* |
| **Ft** | –0.053 *0.7* | **0.29   *0.034*** | 0.18 *0.19* | **0.41 *0.002*** | 0.19 *0.17* | 0.23 *0.095* | 0.1   *0.46* | –0.17   *0.24* |
| **Tf** | 0.2   *0.16* | –0.17   *0.22* | –0.11   *0.42* | –0.066 *0.64* | 0.005 *0.97* | –0.13   *0.36* | 0.23 *0.097* | –0.075 *0.59* |
| **Tf saturation** | 0.074 *0.6* | 0.038 *0.79* | –0.04   *0.78* | 0.078 *0.58* | –0.045 *0.75* | 0.15 *0.29* | –0.074 *0.6* | 0.054 *0.7* |
| **Tf receptors** | 0.029 *0.84* | 0.15 *0.29* | **–0.31   *0.025*** | –0.19   *0.18* | –0.13   *0.34* | –0.28   *0.039* | 0.1   *0.45* | –0.12   *0.37* |
| ***Correlations with PCS*** | | | | | | | | |
| **Iron** | 0.016 *0.91* | –0.01   *0.94* | 0.05 *0.72* | 0.13 *0.34* | 0.15 *0.27* | 0.17 *0.21* | –0.011 *0.94* | 0.13 *0.33* |
| **Ft** | 0.017 *0.9* | 0.18 *0.2* | 0.24 *0.084* | **0.35 *0.009*** | **0.31 *0.023*** | 0.16 *0.24* | 0.12 *0.39* | –0.053 *0.7* |
| **Tf** | 0.18 *0.2* | –0.071 *0.61* | –0.1     *0.46* | –0.15   *0.29* | –0.19   *0.17* | –0.18   *0.2* | **0.29 *0.037*** | –0.15   *0.27* |
| **Tf saturation** | –0.026 *0.85* | 0.002 0.99 | 0.057 *0.68* | 0.14 *0.3* | 0.19 0.17 | 0.18 *0.18* | –0.12   *0.39* | 0.16 *0.24* |
| **Tf receptors** | 0.031 *0.82* | 0.1   *0.47* | **–0.31   *0.022*** | –0.25   *0.071* | –0.22   *0.1* | –0.22   *0.11* | 0.064 *0.65* | –0.18   *0.19* |
| ***Correlations with*** $R_{2}$ | | | | | | | | |
| **Iron** | 0.078 *0.58* | –0.092 *0.51* | –0.07   *0.62* | 0.088 *0.54* | 0.073 *0.6* | 0.038 *0.79* | –0.13   *0.36* | 0.045 *0.75* |
| **Ft** | 0.14 *0.31* | 0.045 *0.75* | 0.12 *0.4* | 0.21 *0.14* | 0.26 *0.065* | 0.21 *0.13* | 0.002 *0.99* | 0.13 *0.36* |
| **Tf** | 0.11 *0.44* | –0.063 *0.66* | –0.11   *0.46* | –0.12   *0.39* | –0.22   *0.12* | –0.13   *0.34* | 0.064 *0.65* | –0.19   *0.18* |
| **Tf saturation** | 0.031 *0.83* | –0.083 *0.56* | –0.03   *0.83* | 0.096 *0.5* | 0.11 *0.43* | 0.048 *0.74* | –0.17   *0.22* | 0.11 *0.44* |
| **Tf receptors** | –0.17   *0.23* | 0.16 *0.25* | –0.22   *0.13* | –0.18   *0.2* | –0.17   *0.22* | –0.23   *0.11* | 0.06 *0.67* | –0.071 *0.62* |
| ***Correlations with [^11^C]SCH23390 BP_ND_*** | | | | | | | | |
| **Iron** |  |  |  | –0.17   *0.31* | –0.22   *0.19* | –0.11   *0.53* | **–0.33   *0.04*** | **–0.39   *0.016*** |
| **Ft** |  |  |  | **–0.35   *0.032*** | **–0.41   *0.016*** | **–0.25   *0.037*** | **–0.39   *0.015*** | –0.22   *0.18* |
| **Tf** |  |  |  | 0.12 *0.46* | 0.22 *0.19* | 0.007 *0.98* | 0.15 *0.37* | –0.15   *0.38* |
| **Tf saturation** |  |  |  | –0.2     *0.24* | –0.3     *0.07* | –0.066 *0.69* | **–0.39   *0.017*** | –0.23   *0.17* |
| **Tf receptors** |  |  |  | 0.003 *0.99* | 0.074 *0.66* | –0.08   *0.63* | 0.24 *0.16* | 0.3   *0.068* |

**BP_ND_** = non-displaceable binding potential; **Ft** = ferritin; **PCS** = paramagnetic contribution to susceptibility; $R_{2}$ = effective transverse relaxation rate; **RN** = red nucleus; **SN** = substantia nigra; **StN** = subthalamic nucleus; **striatum** = combined putamen and caudate; **Tf** = transferrin; **Δχ** = bulk magnetic susceptibility**.**

**Supplementary Table 3. Correlations of neuroimaging parameters and YGTSS results.** Pearson’s coefficients $R$ (roman font) are presented above uncorrected $P$-values (italic font) for correlations between YGTSS metrics (motor and vocal subscores and total score) and the MRI parameters Δχ, PCS and $R_{2}$ as well as the PET-derived [^11^C]SCH23390 $\text{BP}_{\text{ND}}$ in different subcortical ROIs. The cells marked with grey shading denote significant correlations ($R$> 0.2 and $P$ < 0.05).

|  |  |  |  | **SN** | **StN** | **RN** | **Striatum** | **Putamen** | **Caudate** | **Pallidum** | **Thalamus** |
| --- | --- | --- | --- | --- | --- | --- | --- | --- | --- | --- | --- |
|  |  |  | ***Correlations with Δχ*** | | | | | | | | |
|  |  |  | **YGTSS motor** | 0.028 *0.9* | 0.093 *0.69* | 0.057 *0.81* | 0.041 *0.86* | 0.18 *0.43* | –0.14   *0.56* | –0.027 *0.91* | 0.19 *0.41* |
|  |  |  | **YGTSS vocal** | –0.14   *0.54* | –0.12   *0.6* | –0.14   *0.55* | –0.019 *0.94* | 0.073 *0.75* | –0.12   *0.6* | –0.027 *0.91* | 0.09 *0.7* |
|  |  |  | **YGTSS total** | 0.02 *0.93* | 0.022 *0.92* | –0.18   *0.44* | –0.19   *0.41* | –0.10   *0.65* | –0.33   *0.14* | 0.017 *0.94* | 0.084 *0.72* |
|  |  | ***Correlations with PCS*** | | | | | | | | | |
|  |  | **YGTSS motor** | | 0.13 *0.58* | –0.019 *0.93* | 0.041 *0.86* | 0.057 *0.81* | 0.18 *0.44* | –0.13   *0.56* | 0.037 *0.87* | 0.32 *0.16* |
|  |  | **YGTSS vocal** | | 0.2   *0.37* | –0.22   *0.34* | –0.16   *0.5* | –0.017 *0.94* | 0.11 *0.62* | –0.2     *0.4* | 0.041 *0.86* | 0.01 *0.97* |
|  |  | **YGTSS total** | | 0.093 *0.69* | –0.15   *0.52* | –0.24   *0.29* | –0.19   *0.4* | –0.036 *0.88* | –0.36   *0.11* | 0.053 *0.82* | 0.088 *0.71* |
|  | ***Correlations with*** $R_{2}$ | | | | | | | | | | |
| **YGTSS motor** | | | | 0.43 *0.056* | 0.11 *0.64* | 0.021 *0.93* | 0.047 *0.84* | 0.096 0.69 | –0.077 *0.75* | 0.32 *0.16* | 0.26 *0.2* |
| **YGTSS vocal** | | | | 0.15 *0.52* | –0.13   *0.58* | –0.15   *0.53* | –0.084 *0.72* | 0.011 *0.96* | –0.13   *0.58* | 0.14 *0.56* | –0.025 *0.92* |
| **YGTSS total** | | | | 0.014 *0.95* | –0.081 *0.73* | –0.23   *0.34* | –0.25   *0.29* | –0.13   *0.6* | –0.34   *0.08* | 0.067 *0.78* | –0.034 *0.89* |
| ***Correlations with [^11^C]SCH23390 BP_ND_*** | | | | | | | | | | | |
| **YGTSS motor** | | | |  |  |  | **–0.55   *0.02*** | **–0.49   *0.05*** | **–0.71   *0.002*** | –0.26   *0.33* | –0.11   *0.68* |
| **YGTSS vocal** | | | |  |  |  | 0.25 *0.35* | 0.17 *0.52* | 0.26 *0.34* | –0.35   *0.18* | –0.26   *0.33* |
| **YGTSS total** | | | |  |  |  | 0.015 *0.96* | 0.046 *0.87* | –0.08   *0.77* | –0.38   *0.14* | –0.3     *0.26* |

**BP_ND_** = non-displaceable binding potential; **PCS** = paramagnetic contribution to susceptibility; $R_{2}$ = effective transverse relaxation rate; **RN** = red nucleus; **SN** = substantia nigra; **StN** = subthalamic nucleus; **striatum** = combined putamen and caudate; **YGTSS** = Yale Global Tic Severity Scale; **Δχ** = bulk magnetic susceptibility.

**Supplementary Table 4. Correlations of neuroimaging parameters and ATQ and PUTS results.** Pearson’s coefficients $R$ (roman font) are presented above uncorrected $P$-values (italic font) for correlations with the MRI parameters Δχ, PCS and $R_{2}$ as well as the PET-derived [^11^C]SCH23390 $\text{BP}_{\text{ND}}$ in different subcortical ROIs.

|  | |  | | **SN** | | **StN** | | **RN** | | **Striatum** | | **Putamen** | | **Caudate** | | **Pallidum** | **Thalamus** |
| --- | --- | --- | --- | --- | --- | --- | --- | --- | --- | --- | --- | --- | --- | --- | --- | --- | --- |
|  | | ***Correlations with Δχ*** | | | | | | | | | | | | | | | |
| **ATQ  motor** | | **Frequency** | | –0.33   *0.16* | | –0.2     *0.42* | | 0.21 *0.38* | | –0.11   *0.66* | | 0.013 *0.96* | | –0.039 *0.87* | | –0.21   *0.38* | 0.08 *0.74* |
|  |  | **Intensity** | | –0.38   *0.11* | | –0.12   *0.63* | | 0.19 *0.43* | | –0.043 *0.86* | | 0.091 *0.71* | | 0.083 *0.73* | | –0.18   *0.45* | 0.038 *0.88* |
| **ATQ  vocal** | | **Frequency** | | 0.18 *0.46* | | 0.051 *0.84* | | –0.026 *0.92* | | 0.19 *0.44* | | 0.19 *0.43* | | 0.042 *0.86* | | 0.048 *0.84* | 0.088 *0.72* |
|  |  | **Intensity** | | 0.24 *0.32* | | 0.054 *0.83* | | 0.035 *0.89* | | 0.078 *0.75* | | 0.17 *0.49* | | 0.018 *0.94* | | –0.063 *0.8* | 0.085 *0.73* |
| **PUTS** | | | | –0.11   *0.65* | | 0.23 *0.34* | | –0.058 *0.81* | | 0.083 *0.73* | | 0.085 0.73 | | 0.099 *0.69* | | 0.27 *0.27* | –0.11   *0.65* |
|  | | ***Correlations with PCS*** | | | | | | | | | | | | | | | |
| **ATQ  motor** | | **Frequency** | | –0.32   *0.18* | | –0.26   *0.28* | | 0.23 *0.35* | | –0.095 *0.7* | | –0.002 *0.99* | | –0.058 *0.81* | | –0.23   *0.34* | 0.26 *0.28* |
|  |  | **Intensity** | | –0.37   *0.12* | | –0.14   *0.57* | | 0.13 *0.59* | | –0.013 *0.96* | | 0.053 *0.83* | | 0.044 *0.86* | | –0.17   *0.48* | 0.16 *0.51* |
| **ATQ  vocal** | | **Frequency** | | 0.18 *0.45* | | 0.011 *0.96* | | 0.085 *0.73* | | 0.2   *0.42* | | 0.3   *0.22* | | –0.031 *0.9* | | 0.021 *0.93* | 0.047 *0.85* |
|  |  | **Intensity** | | 0.27 *0.27* | | 0.017 *0.94* | | 0.031 *0.9* | | 0.11 *0.67* | | 0.22 *0.37* | | –0.023 *0.93* | | –0.07   *0.78* | 0.007 *0.98* |
| **PUTS** | | | | –0.13   *0.58* | | 0.33 *0.17* | | –0.21   *0.4* | | 0.091 *0.71* | | 0.023 *0.93* | | 0.099 0.69 | | 0.21 *0.38* | –0.12   *0.63* |
|  | | ***Correlations with*** $R_{2}$ | | | | | | | | | | | | | | | |
| **ATQ  motor** | | **Frequency** | | –0.15   *0.54* | | 0.068 *0.79* | | 0.3   *0.23* | | –0.074 *0.77* | | 0.032 *0.9* | | –0.2     *0.42* | | –0.36   *0.14* | 0.37 *0.14* |
|  |  | **Intensity** | | –0.22   *0.38* | | 0.16 *0.53* | | 0.21 *0.4* | | 0.024 *0.92* | | 0.099 *0.7* | | –0.11   *0.68* | | –0.12   *0.63* | 0.25 *0.31* |
| **ATQ  vocal** | | **Frequency** | | 0.079 *0.75* | | 0.045 *0.86* | | 0.18 *0.46* | | 0.13 *0.62* | | 0.24 *0.33* | | –0.13   *0.61* | | –0.004 *0.99* | 0.26 *0.3* |
|  |  | **Intensity** | | 0.11 *0.66* | | 0.12 *0.65* | | 0.099 *0.7* | | 0.005 *0.98* | | 0.14 *0.57* | | –0.27   *0.28* | | 0.032 *0.9* | 0.2   *0.44* |
| **PUTS** | | | | –0.17   *0.49* | | 0.3   *0.23* | | –0.2     *0.42* | | 0.046 *0.85* | | –0.005 *0.98* | | –0.056 *0.82* | | 0.33 *0.18* | –0.19   *0.46* |
|  | ***Correlations with [^11^C]SCH23390 BP_ND_*** | | | | | | | | | | | | | | | | |
| **ATQ motor** | **Frequency** | |  | |  | |  | | –0.026 *0.93* | | –0.023 *0.93* | | –0.018 *0.95* | | –0.2     *0.47* | | –0.077 *0.79* |
|  | **Intensity** | |  | |  | |  | | –0.17   *0.54* | | –0.11   *0.69* | | –0.21   *0.45* | | –0.3     *0.29* | | –0.11   *0.71* |
| **ATQ vocal** | **Frequency** | |  | |  | |  | | 0.14 *0.62* | | 0.071 *0.8* | | 0.29 *0.30* | | –0.18   *0.52* | | 0.089 *0.75* |
|  | **Intensity** | |  | |  | |  | | 0.074 *0.79* | | 0.024 *0.93* | | 0.18 *0.52* | | –0.33   *0.11* | | –0.15   *0.6* |
| **PUTS** | | |  | |  | |  | | –0.072 *0.8* | | 0.04 *0.89* | | –0.16   *0.56* | | 0.13 *0.65* | | –0.17   *0.55* |

**ATQ** = Adult Tic Questionnaire; **BP_ND_** = non-displaceable binding potential; **PCS** = paramagnetic contribution to susceptibility; **PUTS** = Premonitory Urge for Tics Scale; $R_{2}$ = effective transverse relaxation rate; **RN** = red nucleus; **SN** = substantia nigra; **StN** = subthalamic nucleus; **striatum** = combined putamen and caudate; **Δχ** = bulk magnetic susceptibility**.**


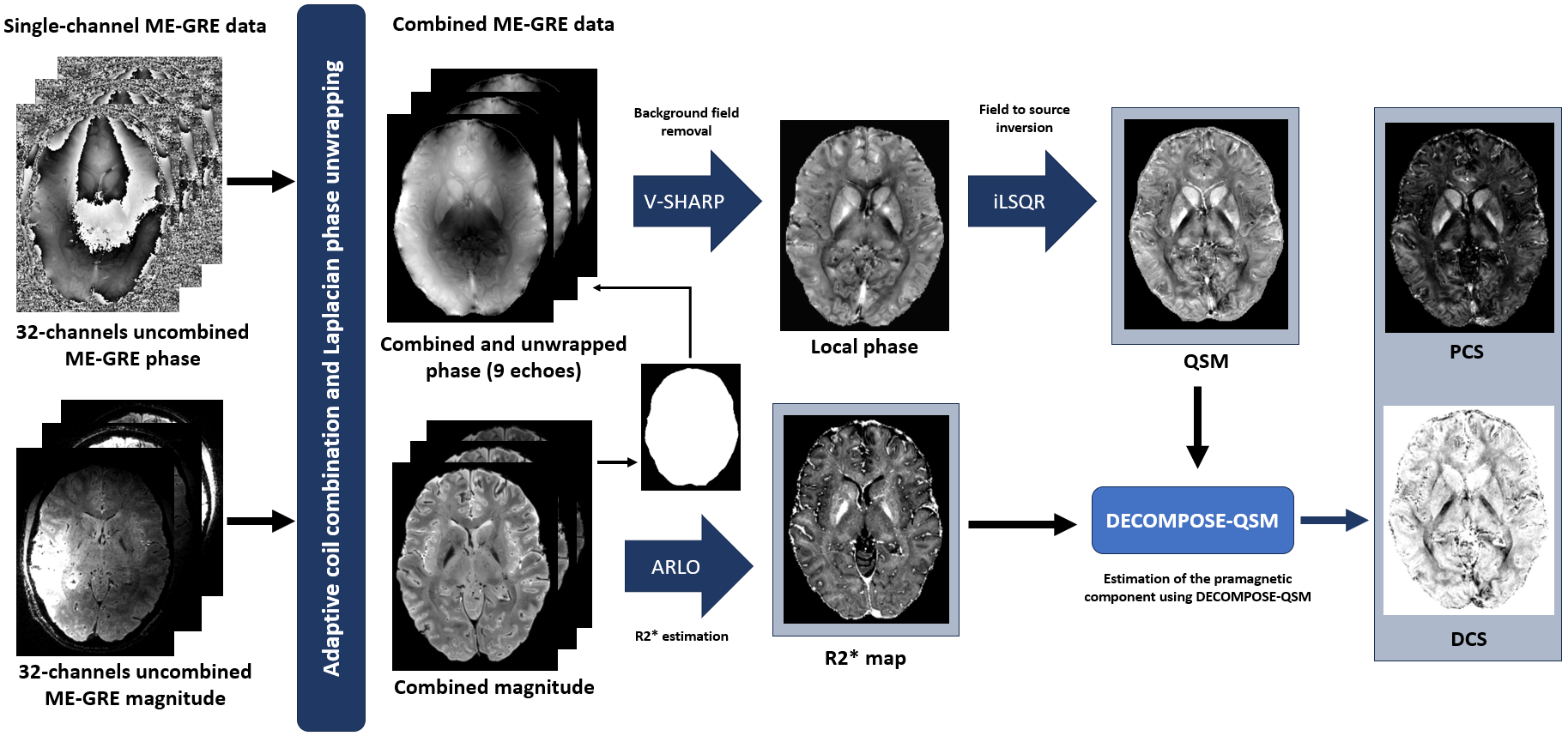


**Supplementary Figure 1. Simplified schematic of the steps involved in generating various maps of MRI-based iron surrogates.** Included are $R_{2}$ maps, QSM and PCS maps (highlighted by light blue shading). More details of the individual steps are given in the main text.


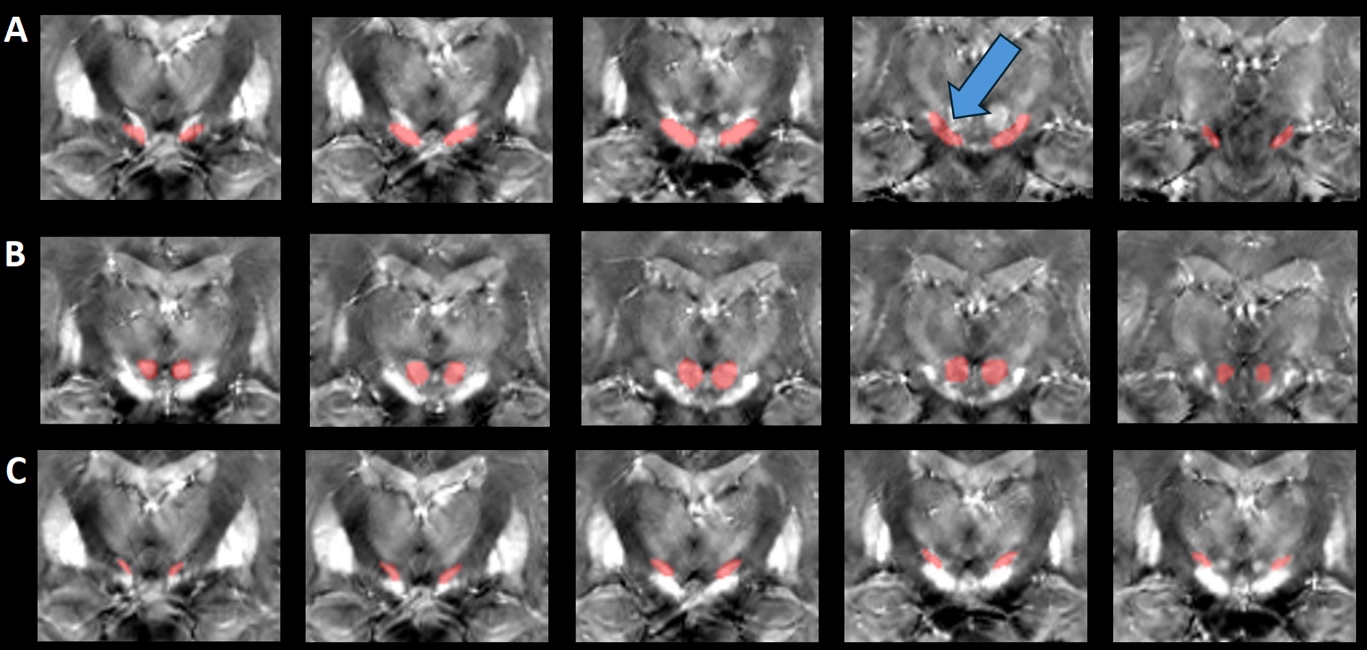


**Supplementary Figure 2. Zoomed-in view of subcortical parcellations. (A)** Curvilinear structure of the substantia nigra (SN), **(B)** oval-shaped, iron-rich red nucleus (RN), and **(C)** subthalamic nucleus (StN), all projected on coronal QSM maps (from rostral to caudal direction). In the SN, hypointensity—characteristic of nigrosome 1—is evident on slices number 5 and 6 (indicated by a blue arrow). No differentiation was made between the SN subregions, pars compacta and pars recticulata, as higher spatial resolution would be required for proper identification**.**


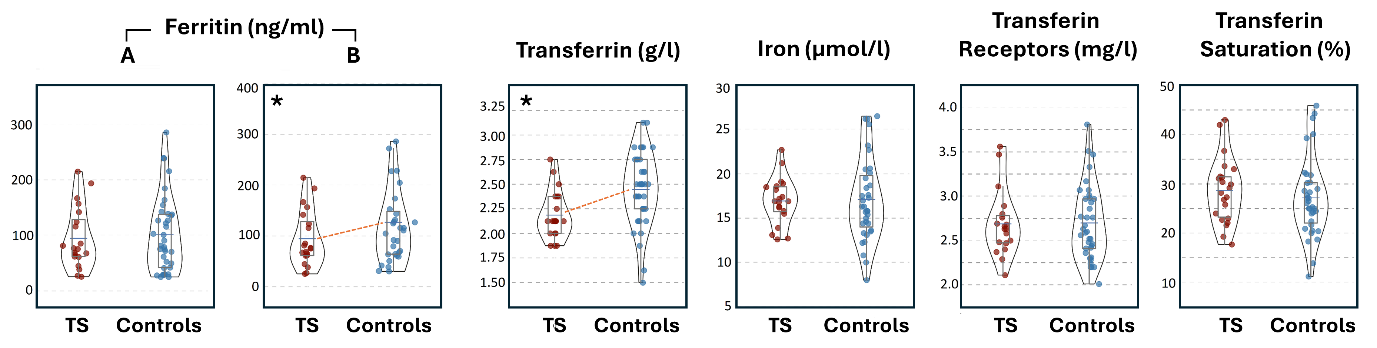


**Supplementary Figure 3. Differences between patients (red symbols;** $N$**=21) and controls (blue symbols;** $N$**=35) in blood iron measures.** Included are results (Mean±SD and Welch’s $t$-tests) for (from left to right) ferritin [**(A)** comparison of all TS patients and control subjects (94±53 ng/ml vs. 95±67 ng/ml, $t$= –0.25, $P$=0.79); **(B)** same comparison after removing subjects with recently adjusted eating behavior towards a low-meat, vegetarian or vegan diet (95±53 ng/ml vs. 126±88 ng/ml, $t$= –1.58, $P$=0.05)], transferrin (2.34±0.18 g/l vs. 2.56±0.29 g/l, $t$= –3.30, $P$=0.001), serum iron (17.0±3.2 µmol/l vs. 17.7±5.0 µmol/l, $t$= –0.16, $P$=0.86), soluble transferrin receptors (2.68±0.35 mg/l vs. 2.69±0.41 mg/l, $t$=0.47, $P$=0.23), and transferrin saturation (28.6±6.5% vs. 27.2±7.8%, $t$=0.75, $P$=0.45).


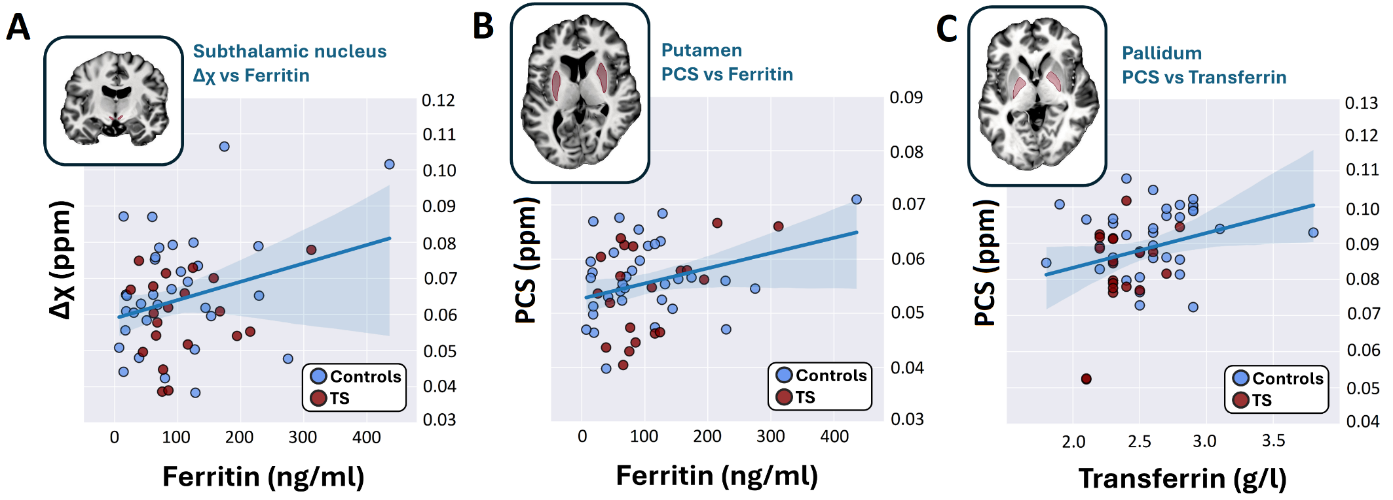


**Supplementary Figure 4**. **Linear relations** $y=ax+b$ **of MRI-derived brain iron surrogates and serum iron metrics.** Variation of **(A)** $\Delta\chi$ in StN and serum ferritin ($a$=5.1×10^–5^ ppm/(ng/ml), $b$=0.058 ppm), of **(B)** PCS in putamen and serum ferritin ($a$=2.8×10^–5^ ppm/(ng/ml), $b$=0.052 ppm), and of **(C)** PCS in pallidum and serum transferrin ($a$=0.0096 ppm/(ng/ml), $b$=0.064 ppm) in the combined cohort of patients (blue symbols; $N$=21) and controls (red symbols; $N$=35). Data from one patient was excluded due to a presumably corrupted ferritin measurement. Further analyses yielded $R$=0.29, $P$=0.03 for the correlation of $\Delta\chi$ in StN and serum ferritin; $R$=0.31, $P$=0.02 for the correlation of PCS in putamen and serum ferritin; and $R$=0.29, $P$=0.03 for the correlation of PCS in pallidum and serum transferrin. All correlations were assessed by calculating Pearson’s coefficient $R$.


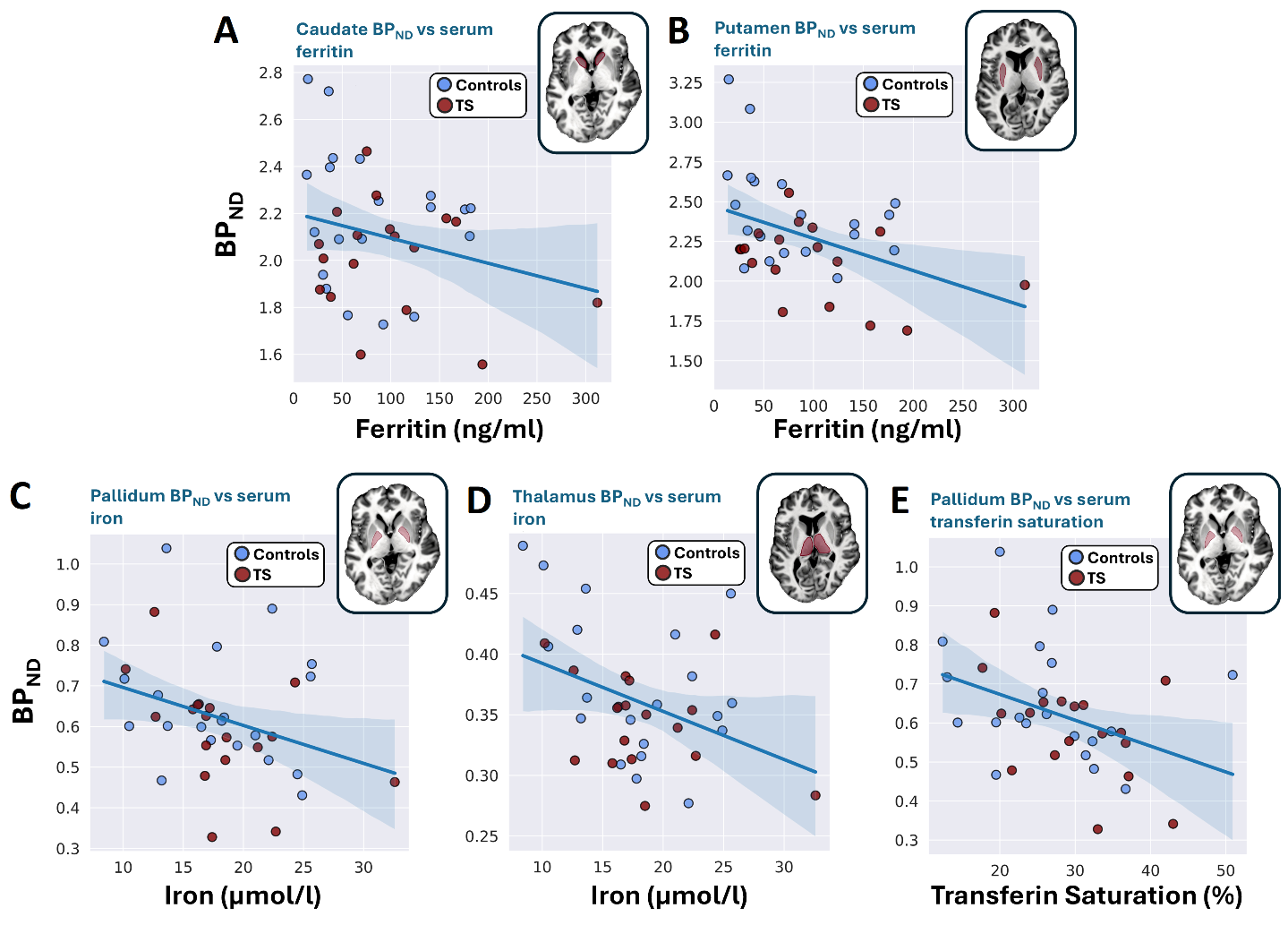


**Supplementary Figure 5. Selected linear relations** $y=ax+b$ **of [^11^C]SCH23390 BP_ND_ and serum iron metrics.** Variations of BP_ND_ with **(A)** serum ferritin in caudate ($a$= –0.0011, $b$=2.20, $R$= –0.25, $P$=0.037), with **(B)** serum ferritin in putamen ($a$= –0.002, $b$=2.47, $R$= –0.41, $P$=0.016), with **(C)** total serum iron in pallidum ($a$= –0.0093, $b$=0.78; $R$= –0.33, $P$=0.04), with **(D)** total serum iron in thalamus ($a$= –0.0039, $b$=0.43; $R$= –0.39, $P$=0.016), and with **(E)** serum transferrin saturation in pallidum ($a$= –0.0066, $b$=0.81; $R$= –0.39, $P$=0.017) in the combined cohort of patients (red symbols; $N$=18) and controls (blue symbols; $N$=20) that participated in the PET-MRI part of the study. All correlations were assessed by calculating Pearson’s coefficient.


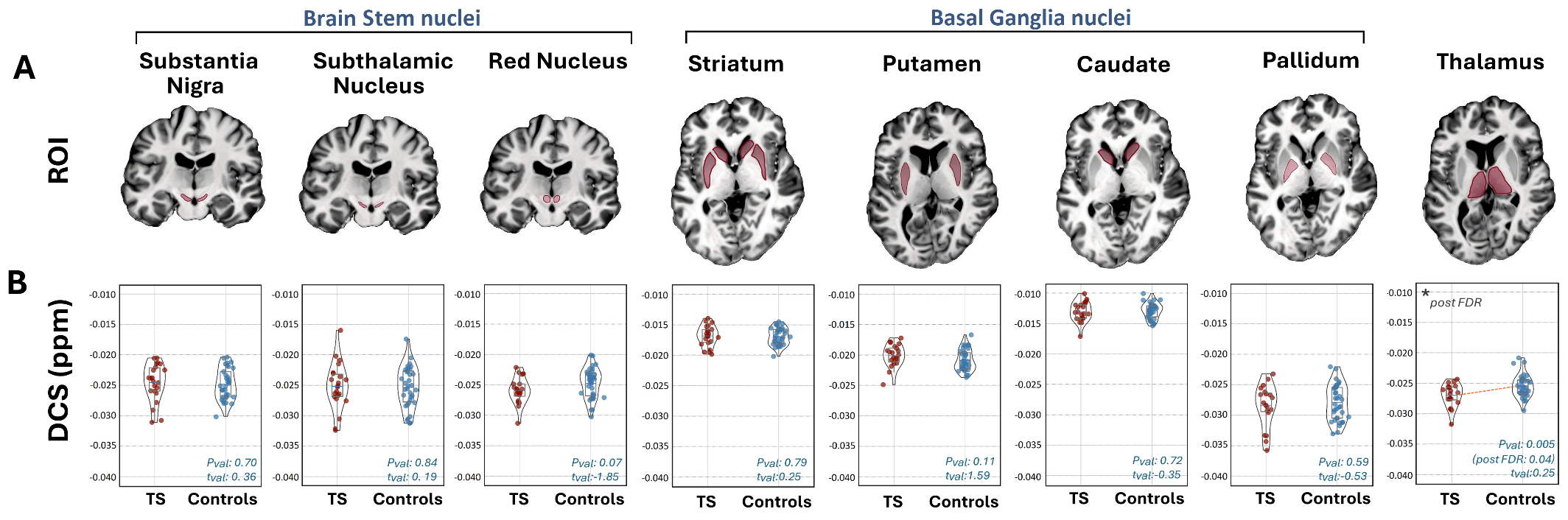


**Supplementary Figure 6. Contributions from diamagnetic sources to the local bulk susceptibility in subcortex.** Shown are corresponding ROIs indicated on a representative slice of the fourth echo of the ME-GRE volumes **(A)** as well as violin plots of regional DCS differences between patients with TS (red symbols; $N$=21) and control subjects (blue symbols; $N$=35) **(B)**. Two-sided Welch’s $t$-test results indicate that the DCS values were slightly increased (i.e., towards “less negative” susceptibility) in the thalamus. All other differences were insignificant already before FDR correction. This further supports the interpretation that the differences observed with QSM were driven by the local iron content and not due to potential myelination differences.
